# DNA strand-exchange patterns associated with double-strand break-induced and spontaneous mitotic crossovers in *Saccharomyces cerevisiae*

**DOI:** 10.1371/journal.pgen.1007302

**Published:** 2018-03-26

**Authors:** Yee Fang Hum, Sue Jinks-Robertson

**Affiliations:** Department of Molecular Genetics and Microbiology and the University Program in Genetics and Genomics, Duke University, Durham, North Carolina, United States of America; The University of North Carolina at Chapel Hill, UNITED STATES

## Abstract

Mitotic recombination can result in loss of heterozygosity and chromosomal rearrangements that shape genome structure and initiate human disease. Engineered double-strand breaks (DSBs) are a potent initiator of recombination, but whether spontaneous events initiate with the breakage of one or both DNA strands remains unclear. In the current study, a crossover (CO)-specific assay was used to compare heteroduplex DNA (hetDNA) profiles, which reflect strand exchange intermediates, associated with DSB-induced *versus* spontaneous events in yeast. Most DSB-induced CO products had the two-sided hetDNA predicted by the canonical DSB repair model, with a switch in hetDNA position from one product to the other at the position of the break. Approximately 40% of COs, however, had hetDNA on only one side of the initiating break. This anomaly can be explained by a modified model in which there is frequent processing of an early invasion (D-loop) intermediate prior to extension of the invading end. Finally, hetDNA tracts exhibited complexities consistent with frequent expansion of the DSB into a gap, migration of strand-exchange junctions, and template switching during gap-filling reactions. hetDNA patterns in spontaneous COs isolated in either a wild-type background or in a background with elevated levels of reactive oxygen species (*tsa1*Δ mutant) were similar to those associated with the DSB-induced events, suggesting that DSBs are the major instigator of spontaneous mitotic recombination in yeast.

## Author summary

Chromosome breakage during mitosis is a threat to genome stability, and duplex integrity can be restored through the process of homologous recombination. Although double-strand breaks (DSBs) are a potent initiator of mitotic recombination, their role in the initiation of spontaneous events is less clear. During recombination, single strands of the broken molecule pair with the complementary strands of an intact repair template to form heteroduplex (het) DNA, which is a key intermediate in the repair process. The canonical DSB repair model makes specific predictions about where hetDNA forms relative to an initiating break, and these predictions were tested using a budding yeast system that detects crossing over between different chromosomes. Following the introduction of a defined DSB, only ~60% of crossovers had the two-sided hetDNA pattern predicted by the model and additional complexities consistent with metastable intermediates were frequent. To determine whether DSBs are the major initiator of spontaneous recombination events, hetDNA patterns associated with spontaneous events were analyzed. These were very similar to those observed following the repair of a defined DSB, suggesting that most spontaneous recombination is initiated by the breakage of both DNA strands.

## Introduction

DNA is constantly assaulted by endogenous damaging agents and maintenance of its integrity is essential for the stable inheritance of genetic material. Double-strand breaks (DSBs), which disrupt both DNA strands, are especially toxic lesions whose repair can result in local sequence changes as well as structural alterations. DSBs can be repaired by homologous recombination (HR), which utilizes an intact duplex as a repair template and is considered a high-fidelity repair mechanism. Alternatively, DSBs can be repaired by the nonhomologous end-joining (NHEJ) pathway, which processes and directly ligates the broken ends and is error prone relative to HR (reviewed by [1]). Single-strand breaks (SSBs), such as nicks and gaps, affect only one strand of DNA and are orders of magnitude more abundant than DSBs [2]. SSBs are natural intermediates during lagging-strand replication, during excision-repair reactions, and during topoisomerase-mediated relief of DNA torsional stress.

Spontaneous recombination repairs random DNA strand breaks that occur during normal cellular metabolism and growth, and DSBs are widely assumed to be the major initiator. This assumption partially reflects the facts that Spo11- and HO-generated DSBs in *Saccharomyces cerevisiae* are physiological triggers for meiotic recombination and mitotic mating-type switching, respectively (reviewed by [3–5]). In addition, DSBs stimulate the integration of transforming DNA [6] and targeted HO or I-*Sce*I DSBs efficiently induce chromosomal HR [7]. Finally, analysis of mitotic loss of heterozygosity in diploids suggest that most initiates with a DSB that occurs prior to chromosomes duplication [8]. Before being supplanted by current DSB-centric HR models [9], however, the early Holliday and Meselson-Radding models proposed that recombination is initiated by a nick [10,11]. There is no doubt that induced DSBs initiate HR, and there have been parallel attempts to assess the recombination potential of targeted SSBs. SSB-stimulated HR in yeast has been examined using sequence-specific nicks introduced by gene II of bacteriophage f1 [12,13] and in mammalian cells using RAG mutant proteins that produce nicks rather than DSBs [14]. Single-strand gaps also have been shown to trigger immunoglobulin gene conversion in the chicken DT40 B cell line [15]. Because SSBs are converted to DSBs during replication, however, it has remained unclear whether nicks/gaps directly trigger HR or must first be converted into a DSB. At least in the specific context of replication, however, a recombination-based template switch to the sister chromatid is an important, error-free gap-filling mechanism for bypassing replication-blocking lesions [16–18]. Finally, it has been argued that lesions other than DSBs (presumably SSBs) are the major initiator of spontaneous mitotic HR in yeast [19]. The most compelling evidence comes from a class of *rad52* mutants that cannot repair ionizing radiation-induced damage or defined DSBs, but remain proficient for spontaneous HR [20]. There recently has been renewed interest in the recombinogenic potential of SSBs in genome editing because these lesions are not substrates for error-prone NHEJ, rendering them less mutagenic than DSBs [21–23]. Recent studies using yeast and mammalian cells have demonstrated that SSBs generated by an I-*Sce*I, I-*Ani*I or CRISPR/CAS9^D10A^ nickase variant can be directly repaired by a non-canonical pathway that uses single-stranded DNA [21,23–25].

In aerobic cells, oxygen metabolism produces reactive oxygen species (ROS) that are a significant source of spontaneous DNA lesions [26]. To counteract the deleterious effects of ROS, cells have evolved multiple ROS-scavenging mechanisms. The yeast *TSA1* gene and its human counterpart, for example, encode a key peroxiredoxin that scavenges endogenously produced hydrogen peroxide [27]. Deletion of *TSA1* is associated with elevated mutagenesis as well as genome rearrangements [28,29]. Furthermore, *TSA1* loss is synthetic lethal with that of genes whose products are required for mitotic recombination [30,31], emphasizing the critical importance of HR in the repair of oxidative damage-induced DNA strand breaks. The precise nature of the damage that triggers ROS-initiated HR is not known, although hydrogen peroxide has been estimated to produce three orders of magnitude more SSBs than DSBs [32]. In addition, whether the pathological DNA strand breaks that initiate spontaneous HR are the same in cells with proficient *versus* defective ROS-scavenging ability is not known.

Heteroduplex DNA (hetDNA) is a key recombination intermediate formed by the pairing of single strands from different duplexes, and its disposition in reciprocal crossover (CO) products is expected to differ depending on whether an event initiates with a DSB or SSB. In the current study, we characterized hetDNA tracts associated with DSB-induced crossing over between ectopic substrates and compared these to those generated during spontaneous crossing over. The canonical DSB repair model predicts formation of a continuous patch of hetDNA on each side of an initiating break, which reflects resection of the broken ends. Furthermore, one of the hetDNA tracts should be present in each CO product and these tracts should be on opposite sides of the break. In addition to frequent tract disruptions, the predicted two-sided hetDNA pattern was observed in only 60% of CO products. The frequent occurrence of one-sided tracts suggests an alternative mode of CO production in which processing of a strand-invasion intermediate occurs prior to extension of the invading end. This leads to breakage of the donor repair template and formation of one CO product; the reciprocal CO product can be generated by a mechanism analogous to synthesis-dependent strand annealing. The presence of a similar proportion of two-sided hetDNA tracts among spontaneous COs isolated in either a wild-type or a *tsa1Δ* background is consistent with DSBs as the major initiator of spontaneous HR in yeast.

## Results

### Different hetDNA patterns are predicted for DSB- *versus* nick/gap-initiated CO events

**Fig 1:**
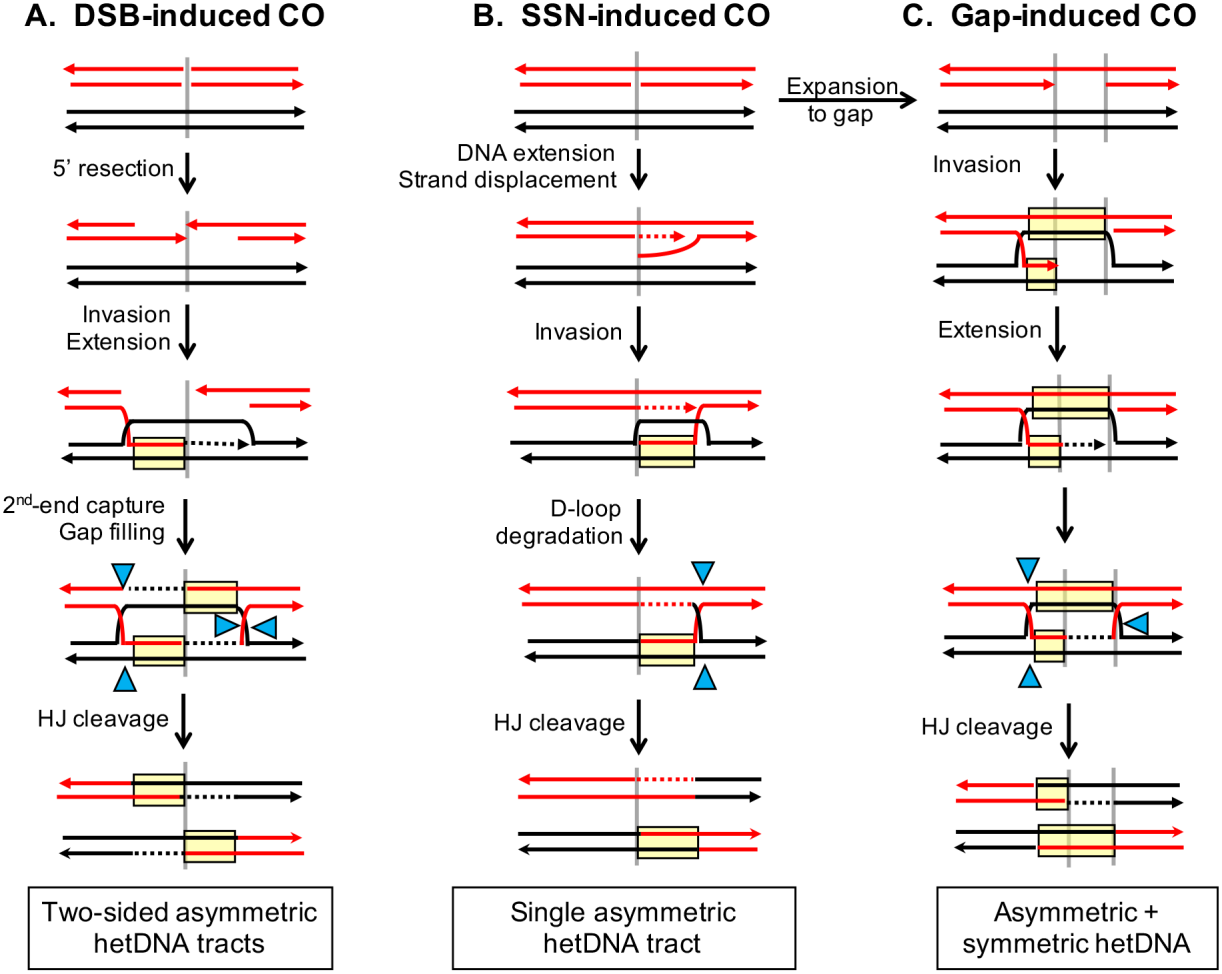
hetDNA patterns predicted for DSB- *versus* nick/gap-induced COs. Solid red and black lines represent the strands of broken and intact chromosomes, respectively. The vertical gray lines indicate the position of the initiating break and arrowheads indicate 3′ ends. Dotted lines correspond to nascent DNA, with the color corresponding to that of the template. hetDNA is boxed in yellow and blue triangles represent HJ cleavage sites. (A) In the classic DSB repair model, 5′ to 3′ resection of each end generates single-strand 3′ tails, one of which invades the donor duplex to create a D-loop and a region of hetDNA. DNA synthesis primed from the invading end enlarges the D-loop until it anneals to the other side of the DSB (2^nd^-end capture), generating a second hetDNA tract and an HJ. Gaps resulting from the initial 5′-end resection are filled prior to cleavage of the HJs by resolvases. There is a hetDNA tract upstream of the DSB in one CO product and a hetDNA tract downstream of the DSB in the other, which is referred to as two-sided asymmetric hetDNA. (B) In the Meselson-Radding model, DNA extension from the 3′ end of a nick displaces a 5′ flap that invades the donor duplex to form a D-loop and a single region of hetDNA. The D-loop is degraded, leading to formation of an HJ. After HJ cleavage, there is a single, asymmetric hetDNA tract within what was originally donor sequence. (C) A nick can be expanded into a gap by an exonuclease, with subsequent formation of adjacent regions of symmetric and asymmetric hetDNA that are flanked by HJs. This hetDNA pattern persists following HJ resolution.

The goals of the current work were (1) to define hetDNA patterns associated with DSB-induced CO events and (2) to use the DSB-associated hetDNA patterns to infer whether spontaneous- and ROS-initiated CO events are most often initiated by DSBs or by single-strand nicks/gaps. Fig 1A presents the formation of CO events *via* the classic model of DSB repair, which initiates when the 5′ ends of the break are resected to generate single-stranded 3′ tails that conduct a homology search (reviewed by [33]). Invasion of a homologous duplex by a 3′ tail creates a region of heteroduplex DNA (hetDNA) in which the invading strand is paired with the complementary strand and the identical strand is single stranded within a displacement (D) loop. Extension from the invading 3′ tail enlarges the D-loop (or the D-loop migrates with the extending end), enabling capture of the 3′ tail on the other side of the DSB to generate a second tract of hetDNA. Following the filling of gaps that reflect the initial 5′-end resection, the hetDNA regions are flanked by two Holliday junctions (HJs). The hetDNA present in this intermediate is defined as “asymmetric” because, at a given location, it is present in only one of the interacting substrates. Following HJ resolution by cleavage/ligation reactions, each CO product is predicted to contain a single hetDNA tract and these should be on opposing sides of initiating break. We refer to this specific hetDNA pattern as “two-sided” asymmetric hetDNA. We previously demonstrated that most DSB-initiated COs in a plasmid-chromosome assay have the distinctive two-sided hetDNA pattern predicted by resolution of HJ-containing intermediates [34].

Although the precise pattern of hetDNA associated with nick/gap-initiated COs cannot be predicted with certainty, it nevertheless is expected to differ from the two-sided pattern predicted for DSB-initiated events (reviewed by [35]). For example, in the early Meselson-Radding model [11], DNA synthesis from the 3′ end of the nick displaces the 5′ end, which then invades the donor duplex to form a single tract of hetDNA (one-sided, asymmetric hetDNA; Fig 1B). Following resolution of the resulting HJ, asymmetric hetDNA is predicted to be present in only one of the CO products, specifically the donor. When the nick is expanded into a single-strand gap by an exonuclease (Fig 1C), each product is expected to have a hetDNA tract at exactly the same position (“symmetric” hetDNA) and only one product to additionally contain a tract of asymmetric hetDNA. Although a tract of symmetric hetDNA can be generated in the DSB repair model if an HJ migrates away from the initiating break, the very distinctive two-sided hetDNA pattern that flanks the break should still persist.

### Mapping hetDNA using a CO-specific chromosomal system

**Fig 2:**
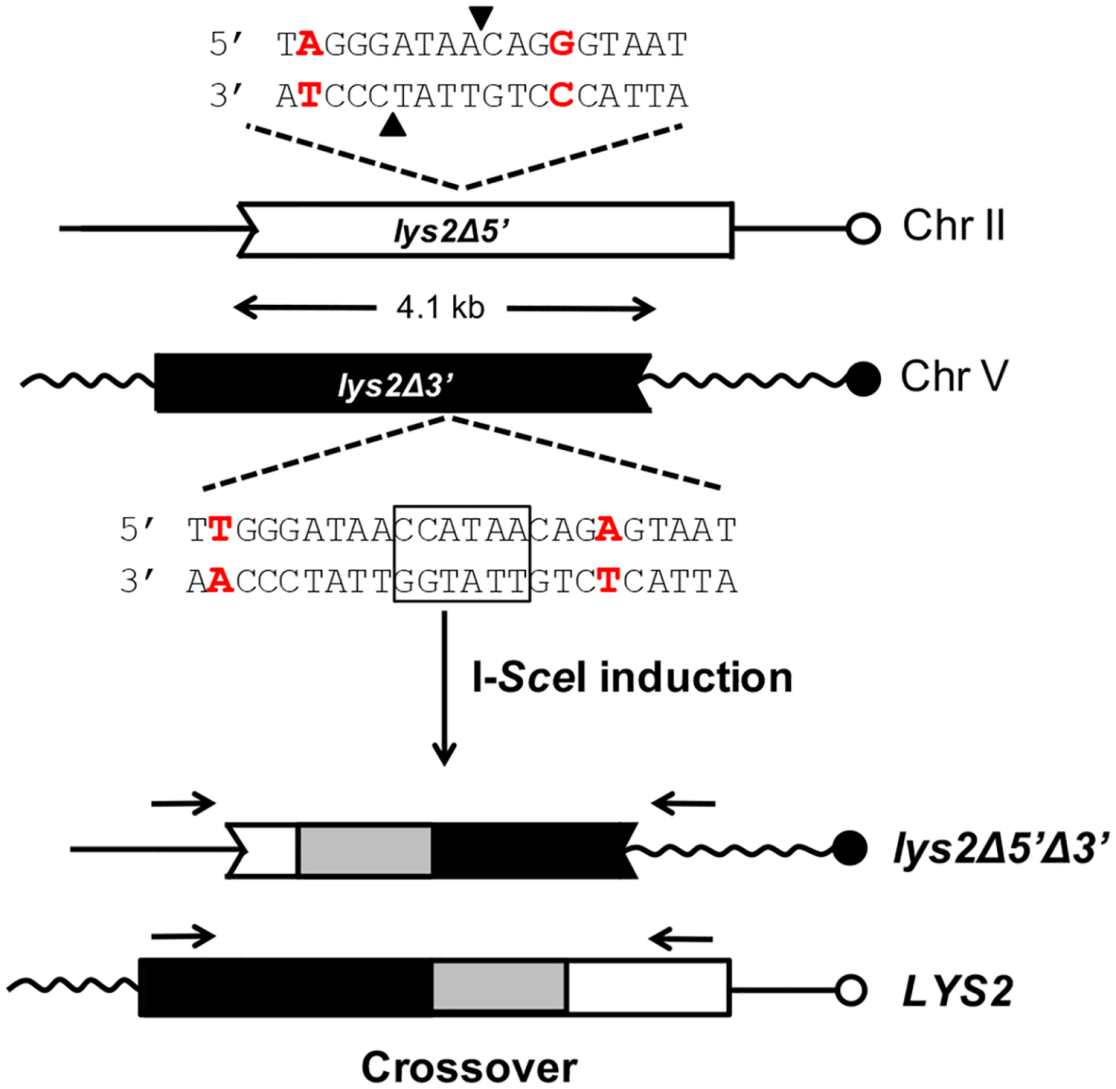
Crossover-specific assay for hetDNA mapping. The recipient *lys2Δ5*′ allele (white box) on chromosome II contains an in-frame I-*Sce*I cut site (arrowheads indicate corresponding nicks). The *lys2Δ3*′ allele (black box) on chromosome V is also in frame and contains an I-*Sce*I cut site inactivated by a 6-bp insertion (boxed). The nearest flanking SNPs (red) are 8 bp from each 3′ end of the I-*Sce*I generated DSB. Only a CO event can reconstitute a full-length *LYS2* allele and both recombination products must co-segregate for Lys^+^ colony formation. hetDNA flanking the initiating DSB is indicated as gray boxes, and was monitored in each product by SMRT sequencing. The product-specific primers for PCR amplification are indicated by black horizontal arrows.

**Fig 3:**
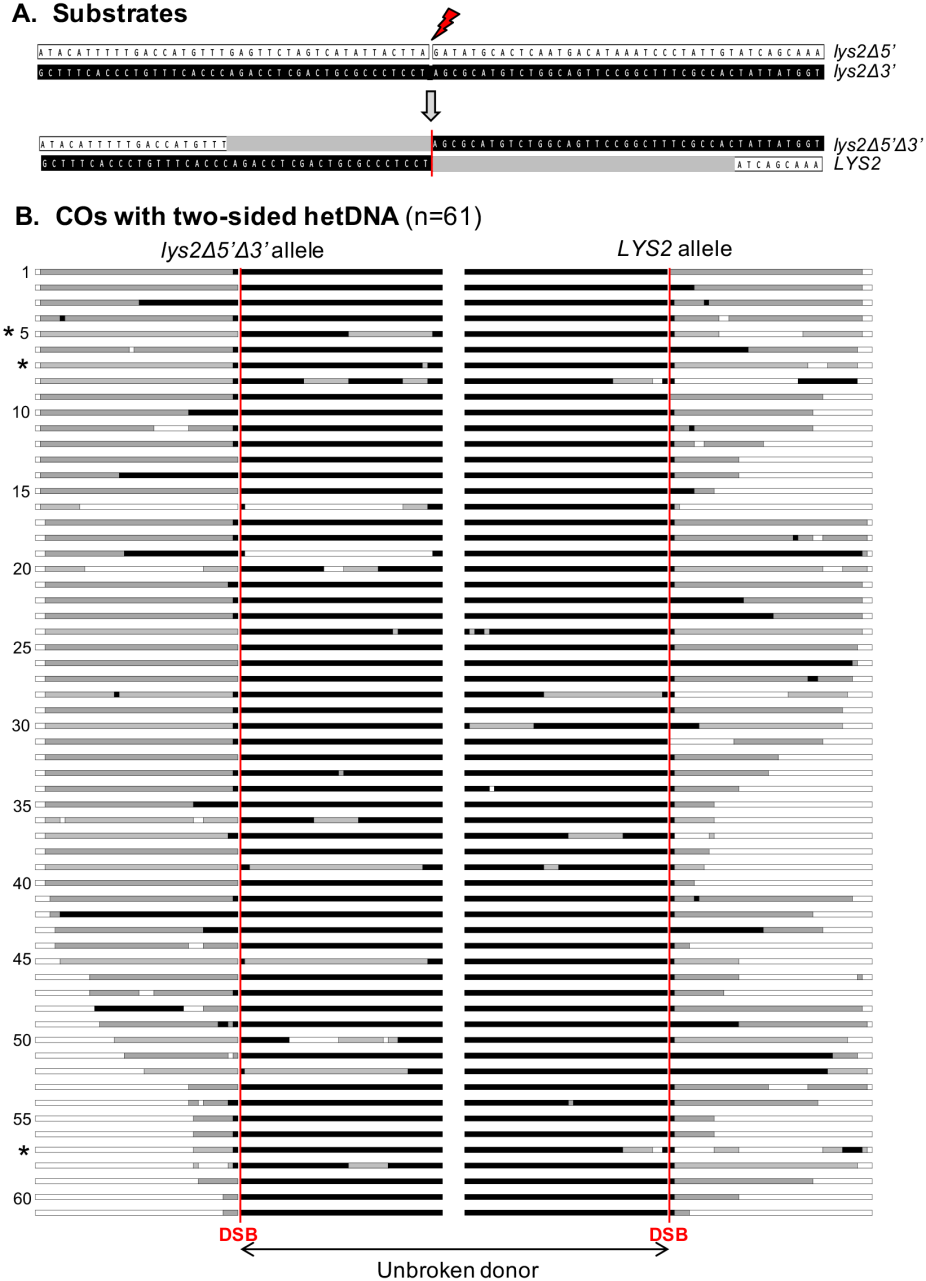
DSB-induced COs with two-sided hetDNA. (A) The *lys2Δ5*′ and *lys2Δ3*′ alleles are shown as white and black boxes, respectively, and the SNPs associated with each are shown. The thunderbolt indicates the position of the DSB in the *lys2Δ5*′ allele. In an idealized DSB-associated CO, the broken white allele receives information from the black allele, generating a single asymmetric hetDNA tract (gray boxes) in each product on opposing sides of the DSB. There is no alteration of the donor black allele. (B) Each line represents the products of individual CO events, with the *lys2Δ5*′*Δ3*′ and *LYS2* alleles in the left and right panels, respectively. The vertical red lines indicate the position of the DSB. In all events, hetDNA was upstream of the DSB in the *lys2Δ5*′*Δ3*′ allele and downstream of the DSB in the *LYS2* allele. The three events marked with an asterisk had terminal symmetric hetDNA consistent with migration of one HJ away from the initiating break (see Fig 4B). “n” is the number of events with the two-sided hetDNA pattern.

The CO-specific system developed for the current study is comprised of truncated *lys2* alleles that share 4.1 kb of homology and are on different chromosomes in a haploid background (Fig 2). One recombination substrate was at the *LYS2* locus on chromosome II; it was truncated at its 5′ end (*lys2Δ5*′ allele) and contained an in-frame I-*Sce*I cut site within a region of the gene that can tolerate amino acid replacements. The repair template was a 3′-truncated allele (*lys2Δ3*′) located at the *CAN1* locus on chromosome V and containing 82 single-nucleotide polymorphisms (SNPs) engineered at ~ 50 bp intervals (Fig 3A and S1 Fig). The donor allele additionally contained an I-*Sce*I cut site inactivated by a 6-bp insertion, which maintains the correct reading frame of the gene. Because the alleles are truncated at different ends, a CO is required to reconstitute a functional, full-length gene. The CO can occur anywhere within the region of homology, and a key feature of the system is that there are no accompanying constraints on either the position or the extent of associated hetDNA. Finally, because co-segregation of the resulting reciprocal translocation products is required for colony growth, both CO products can be analyzed. As in prior studies [34,36], recombinants were isolated in a mismatch repair (MMR)-deficient, *mlh1Δ* background in order to preserve the CO-associated hetDNA footprint. DSB-induced events were isolated following galactose-induced I-*Sce*I expression while spontaneous COs were isolated in a haploid strain that lacked I-*Sce*I. To analyze CO-associated hetDNA, genomic DNA was isolated directly from Lys^+^ colonies and individual recombination products were barcoded during subsequent PCR amplification. The barcoded products were then pooled and sequenced *en masse* using single-molecule real-time (SMRT) technology ([37]; see Methods for details). After sorting sequence reads by barcodes, the presence of both donor and recipient SNPs at a given position was diagnostic of hetDNA.

### DSB-initiated chromosomal COs with two-sided hetDNA

As previously noted, DSB-induced CO products are expected to contain two hetDNA tracts (see Fig 1A), each of which reflects engagement of a broken end with the donor repair template. At least one tract results from strand invasion; the second tract can reflect either an annealing reaction (D-loop capture) or a second strand-invasion event. Because it is the recipient allele that suffers the DSB, only recipient information adjacent to the break is expected to be replaced with hetDNA. The reciprocal products of 166 I-*Sce*I-induced COs were sequenced and 61 had an asymmetric hetDNA tract in each product (two-sided hetDNA), as predicted by the canonical DSB repair model. In addition, there were 38 COs that had hetDNA limited to a single side of the DSB (one-sided hetDNA). Finally, there were 60 COs that contained only gene conversion tracts, and seven that unexpectedly had hetDNA only in what was originally donor sequence. We consider the latter two classes to be uninformative in terms of overall hetDNA patterns and these will not be further considered. Those COs that contained only gene conversion tracts could reflect the loss of one daughter cell following the first post-recombination round of replication, or resolution of a single HJ by replication. Thus, of the 99 COs with informative hetDNA tracts, about 60% (61/99) contained hetDNA on both sides of the initiating break, while 40% (38/99) had hetDNA on only one side.

The CO events containing two-sided hetDNA are shown in Fig 3B; white rectangles/squares correspond to SNPs in the broken, recipient molecule, black to donor SNPs and gray to regions of hetDNA. The hetDNA tracts in the reciprocal products of each CO event are aligned horizontally (stacked profiles are presented in S2 Fig), with their vertical order reflecting the length of hetDNA upstream of the DSB in the doubly-truncated product on the left. Strikingly, in all of the COs with two-sided hetDNA, the tracts upstream and downstream of the DSB were always in the *lys2Δ5′Δ3′* and *LYS2* alleles, respectively. If the HJs in the predicted DSB-repair intermediate are fully ligated and cleavage is random, the relative positions of the hetDNA in the CO products should also be random. If, however, the HJs remain nicked and resolution is nick-directed (this applies also to a single HJ), then as observed, hetDNA is expected to be upstream of the DSB in the doubly-truncated CO product, and downstream of the break in the full-length product (S3 Fig).

**Fig 4:**
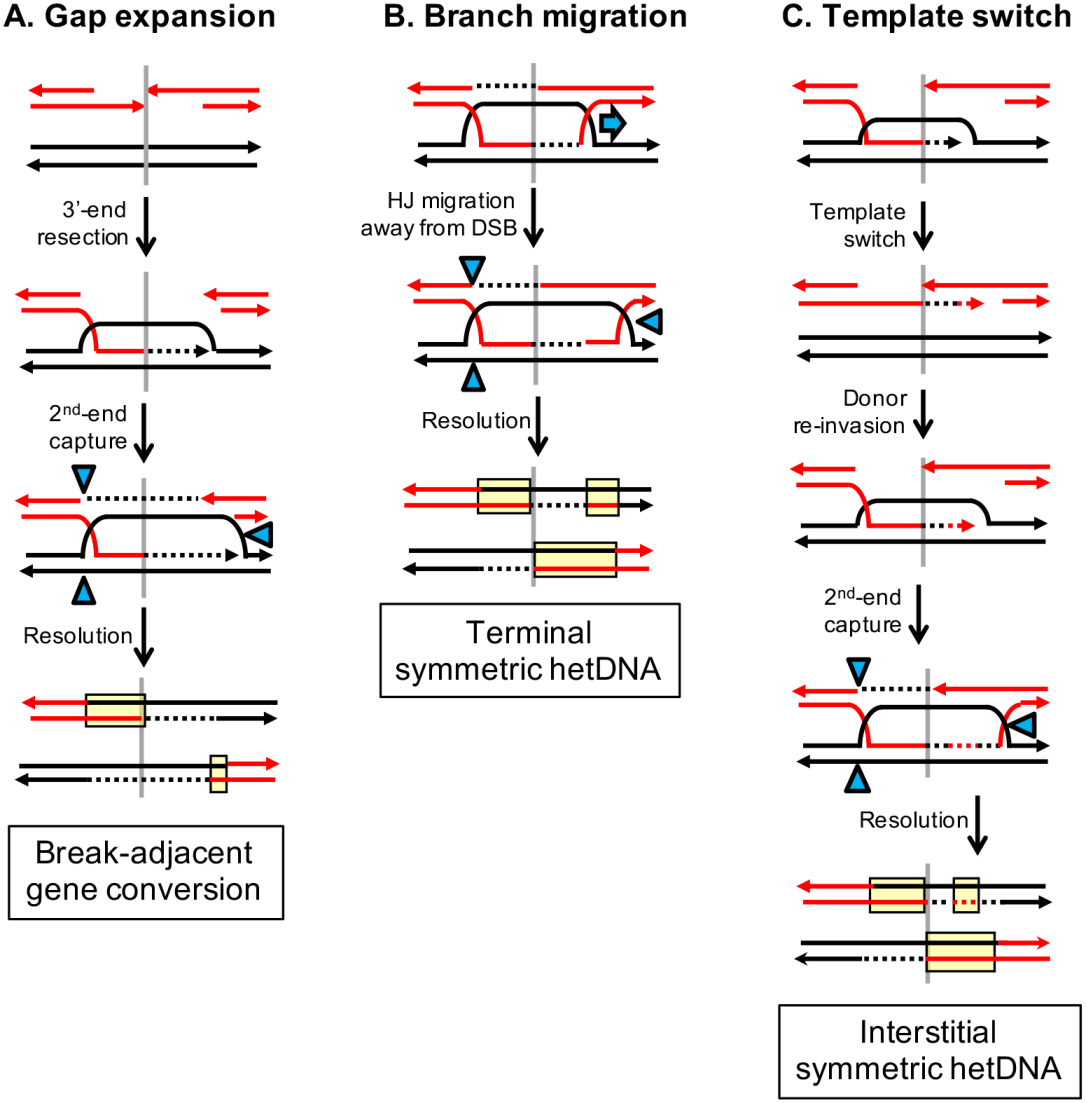
Origins of complexities associated with two-sided hetDNA. Red and black lines represent broken recipient and intact donor sequences, respectively. Yellow boxes correspond to hetDNA and blue-filled triangles to sites of HJ cleavage. The position of the initiating DSB is indicated as a gray, vertical lines. (A) Gap expansion results from loss of the 3′ end of a DSB and produces a gene conversion tract adjacent to the DSB. (B) Branch migration of one HJ away from the DSB creates a tract of terminal symmetric hetDNA. (C) Spontaneous ejection of an extending end from the black donor can be followed by a temporary template switch to the sister chromatid, followed by a final switch back to the donor. This generates an interstitial tract of symmetric hetDNA, the repair of which can result in an interstitial gene-conversion tract.

One feature of CO events that was evident in our previous analysis of non-crossovers (NCOs) in a similar system was the loss of break-proximal recipient SNPs, which produced a tract of apparent gene conversion next to the DSB. These conversion events were of two types: those that removed a single SNP and those that removed a tract of contiguous SNPs. We previously demonstrated that single-SNP removal reflects the exonuclease activity of Pol δ and suggested that multiple-SNP removal reflects gap expansion, which occurs when the 3′ as well as the 5′ end of a break is lost (Fig 4A; [36]). Among the COs with two-sided hetDNA, gap expansion was equally likely on the left (10/61) and right sides (12/61) of the initiating break (p = 0.81). Simultaneous loss of both 3′ overhangs was rare (2/61) and consistent with independent loss of the ends. After eliminating gap expansion events involving multiple SNPs from the CO data, the efficiencies of single-SNP loss were 49% (25/51) upstream and 94% (46/49) downstream of the DSB. The corresponding SNPs generate A:A and C:A mismatches, respectively, when paired with donor sequence (see Fig 2). In the previous analysis of NCO events, the efficiencies of single-SNP removal were reversed, occurring 97% and 47% of the time for the upstream and downstream SNPs, respectively [36]. Because the orientations of the I-*Sce*I cut-site fragments were inverted in the CO-specific assay used here relative to the previous system, the frequency of single-SNP loss tracks with the identity of the terminal mismatch created by strand invasion/annealing. This suggests that the efficiency difference in break-proximal SNP removal primarily reflects the mismatch and/or context specificity of the Pol δ exonuclease activity [36].

Among the 61 COs with two-sided hetDNA, 25% (31/122; hetDNA tracts on each side of the break are considered independent) of tracts were interrupted by interspersed gene conversion and/or restoration. There were 26 restoration and 11 gene conversion events, which corresponds to a bias towards restoration (p = 0.03 by Chi-square). Because experiments were done in the absence of the Mlh1 protein, restoration/conversion tracts presumably reflect mismatch removal that is independent of the canonical MMR system. As noted previously, hetDNA tracts reflect the pairing of strands from donor and recipient duplexes, but they are expected to only be present in segments that correspond to the originally broken molecule. Approximately 20% (23/122) of the sequence originally in the unbroken donor, however, contained tracts of hetDNA and/or gene conversion. The simplest, but rarest (3/23) of these alterations corresponded to terminal, symmetric hetDNA located at the break-distal end of an asymmetric tract (marked with asterisks in Fig 3B). These are most simply explained by migration of an HJ away from the initiating break, which converts asymmetric to symmetric hetDNA (Fig 4B). More often, however, the donor contained a region of interstitial symmetric hetDNA (13/23), gene conversion (2/23) or a combination of both (5/23). Such hetDNA/conversion tracts can be explained by a template switch from the donor to the intact sister of the broken chromatid during 3′-end extension. As illustrated in Fig 4C, this results in an interstitial rather than terminal hetDNA tract; repair of the hetDNA would result either in an interstitial gene conversion tract or undetectable restoration within the repair template.

### DSB-initiated COs with one-sided hetDNA in the recipient

**Fig 5:**
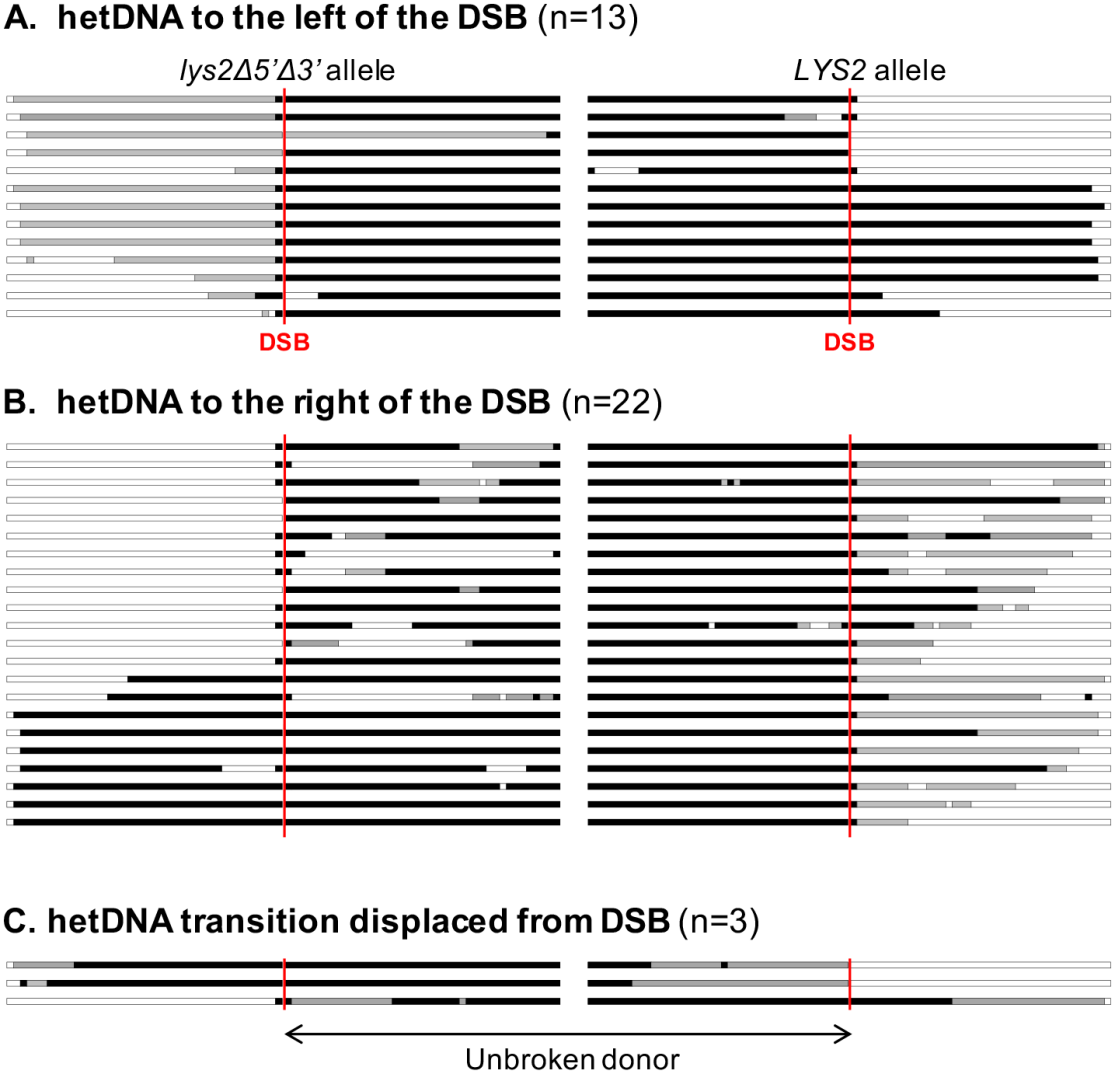
DSB-induced COs with one-sided hetDNA. Each line represents the products of individual CO events, with the *lys2Δ5*′*Δ3*′ allele on the left and the *LYS2* allele on the right; red vertical lines correspond to the position of the initiating DSB. Events with hetDNA limited to the (A) left or (B) right side of the DSB are grouped. (C) The transition of asymmetric hetDNA from one product to the other is displaced from the DSB. “n” is the number of events with the relevant hetDNA pattern.

**Fig 6:**
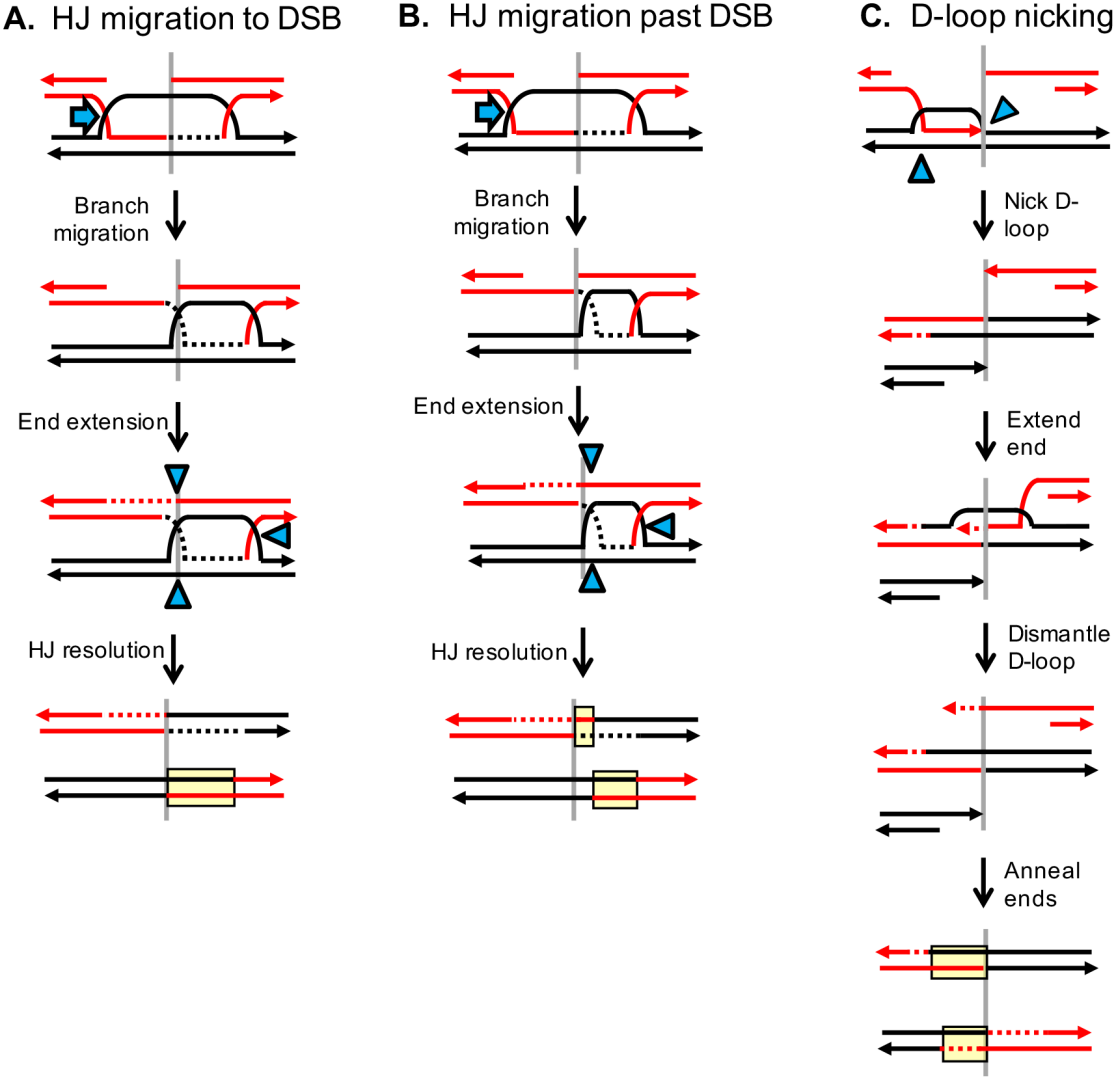
Mechanisms that produce hetDNA on only one side of an initiating DSB. Red and black lines represent recipient and donor sequences, respectively. Yellow boxes correspond to hetDNA and blue-filled triangles to sites of nicking by structure-selective endonucleases. The position of the initiating DSB is indicated by vertical gray lines. (A) Migration of an HJ back to the DSB site prior to second-end extension removes the hetDNA created by the initial strand invasion. (B) Migration of one of the HJs past the site of the DSB after second-end extension places both asymmetric hetDNA tracts on the same side of the break. The transition between the hetDNA tracts remains, but is displaced from break site. (C) The D-loop is nicked prior to extension of the invading end to create a single CO product and a broken arm of the donor chromosome (black). As shown, extension of the 3′ (red) end on the other side of the original DSB is templated from the intact sister chromatid. This provides homology to the broken donor and the reciprocal CO product is generated by an annealing reaction.

Of the 99 reciprocal COs that contained informative hetDNA, 38 had hetDNA on only one side of the initiating break in the products (Fig 5A–5C; see S4 Fig for the stacked profiles). We note that this is the pattern expected of nick-induced COs (Fig 1B), and a formal possibility is that I-*Sce*I frequently makes nicks instead of DSBs in our system. In a nick-induced model, it is specifically the unbroken donor that “receives” a strand from the nicked molecule. Although seven events with donor-limited hetDNA were detected (S5A Fig), all but one also had alterations (gap expansion or gene conversion) to the broken molecule. Of the 38 one-sided tracts, 15 were on the left side of the DSB and 23 were on the right side (p = 0.26). When considered in the context of the canonical DSB repair model, most (35/38) of the one-sided tracts are consistent with (1) very short hetDNA that did not extend far enough to include a SNP, (2) repair of hetDNA on one side of the break as an undetectable restoration event, or (3) helicase-driven migration of the back end of the D-loop to the break site prior to second-end extension (Fig 6A). In the remaining three one-sided events, the transition between the two expected hetDNA tracts was displaced from the break site (Fig 5C; see also S4 Fig). This pattern can be explained by branch migration of one HJ past the original break site so that both junctions are on the same side of the DSB when resolution occurs (Fig 6B).

Although the one-sided hetDNA tracts can be accommodated in the canonical DSB repair model, their high proportion suggests that an alternative CO mechanism might also be operative. An attractive model is one in which there is a single invasion event and D-loop processing occurs prior to the extension of the invading end. This particular model is loosely based on one proposed for the fusion of different donors following their simultaneous invasion by a single 3′ end [38]. As illustrated in Fig 6C, the invaded black donor molecule is nicked at two positions: at the front edge of the D-loop and on the complementary strand at the back edge of the D-loop (blue triangles). The positions of the nicks are modeled after those originally proposed for the Mus81-Eme1 complex during meiotic CO formation in *Schizosaccharomyces pombe* [39]. Ligation of the nicked (top) black strand to the red invading end, together with the filling of the gap created by resection, creates the first of the two CO products. Because both donor strands are nicked prior to extension of the invading end, a black donor arm with a 3’ tail that terminates at (or very near) the position of the I-*Sce*I induced break in the red duplex is generated. The free red and black arms share no homology but can be joined by an SDSA-like event following extension of one of the 3′ ends. As pictured, the only template available for 3′-end extension is the first CO product, although unbroken sister chromatids may also be available. If the red end (produced by I-*Sce*I cleavage) is extended and anneals to the black end (generated by D-loop processing), then hetDNA will only be present on only one side of the initiating DSB. By contrast, extension of the black end and its subsequent annealing to the red end will generate a second hetDNA tract on the other side of the initiating break. This latter product would be indistinguishable from that predicted by canonical DSB repair model.

### Length of hetDNA tracts in DSB-induced COs

As noted above, CO-associated hetDNA tracts were frequently modified by presumptive mismatch correction, branch migration and/or template switching. To estimate the extent of strand pairing between the recombination substrates, we define hetDNA length on each side of the initiating break as the most break-distal transition from heteroduplex to homoduplex DNA, or between donor and recipient sequence. Because many tracts extended to the border of homology, median rather than mean tract lengths were calculated. We also included data from all CO events that contained hetDNA, regardless of hetDNA position(s). On the upstream and downstream sides of the initiating break, the median hetDNA lengths were similar: 1940 bp and 1800 bp, respectively (S1 Appendix). The median total hetDNA length per CO event was 2990 bp, which is somewhat less than the sum of the median hetDNA lengths on both sides (3740 bp).

### hetDNA profiles in spontaneous CO events

The rate of spontaneous recombination in the CO-specific system used here is rare (2 x 10^−8^), which poses a challenge for detecting and analyzing associated hetDNA. In typical recombination experiments, cells are grown in rich liquid medium and spontaneous recombinants are identified by selective plating. Because recombination occurs during non-selective growth, the associated hetDNA footprint will be lost during subsequent cell division in liquid medium. To preserve the footprint, we spotted small numbers of Lys^-^ cells onto rich medium and allowed expansion prior to replica-plating onto selective medium. Each Lys^+^ colony contained the progeny of the cell in which the recombination event occurred, and entire colonies were analyzed using the same sequencing procedure as used for I-*Sce*I-induced recombinants.

**Fig 7:**
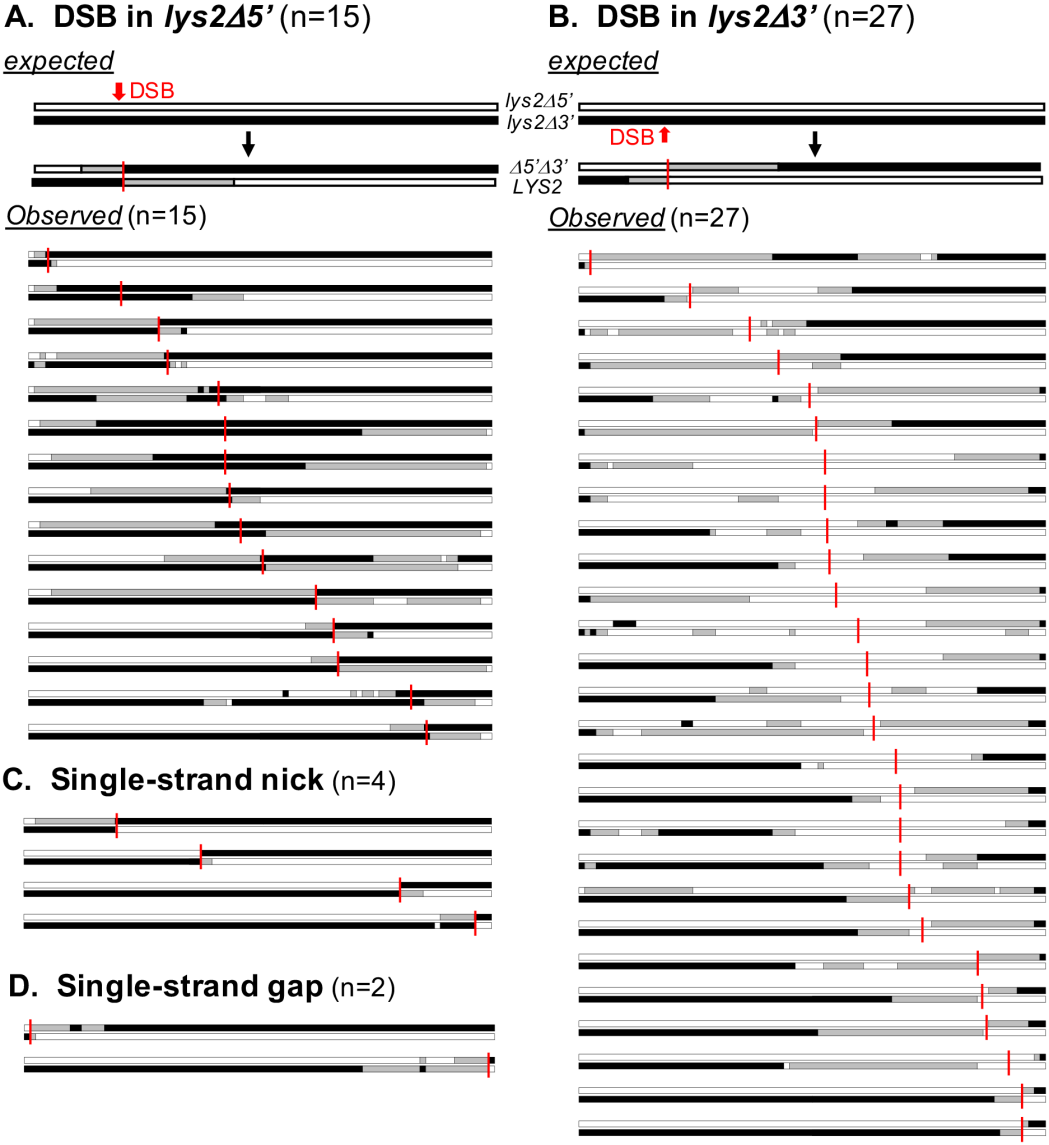
Spontaneous COs in which each product contains hetDNA. White, black, and gray boxes represent *lys2Δ5*′, *lys2Δ3*′ and hetDNA, respectively. Potential initiation sites are marked with vertical red lines. (A) COs with a hetDNA pattern consistent with an initiating DSB in the *lys2Δ5*′ allele. (B) COs with a hetDNA pattern consistent with an initiating DSB in the *lys2Δ3*′ allele. (C) COs exhibiting a hetDNA pattern consistent with initiation by a single-strand nick. (D) COs containing a hetDNA pattern consistent with initiation by a single-strand gap. “n” is the number of events with the relevant hetDNA pattern.

We sequenced 132 spontaneous CO events, of which 77 contained hetDNA; the remaining 55 had only gene conversion tracts. This distribution is very similar to that observed among the DSB-induced COs (106/166 with hetDNA; p = 0.34). Of the 77 COs containing hetDNA, 48 had hetDNA tracts in both products. These profiles are shown in Fig 7, where reciprocal products are stacked to emphasize relative hetDNA positions. Forty-two had the hetDNA pattern predicted of a DSB-induced CO: a transition of asymmetric hetDNA in one CO product to asymmetric hetDNA in the other, sometimes with an intervening region of gene conversion consistent with gap expansion (Fig 7A and 7B). This proportion (42/77) is similar to that of DSB-induced events with the canonical two-sided hetDNA relative to the initiating break (61/106; p = 0.76).

Among the spontaneous COs with the hetDNA pattern predicted of a DSB-initiated event, we inferred which substrate was broken and where the break occurred. In Fig 7, the *lys2Δ5′* and *lys2Δ3′* alleles are indicated as white and black rectangles, respectively, and hetDNA tracts are gray. The minor substrate color in the products corresponds to the broken molecule, and the major color to the repair template. As shown for an expected CO, the hetDNA transition point reflects the position of the initiating break (vertical red line). When there was a region of gene conversion between the asymmetric hetDNA tracts, the break position was arbitrarily placed at the center of the conversion tract, although it likely was at one of the two ends. Using these criteria, there was no initiation bias in the two alleles: 15 events initiated in the *lys2Δ5′* allele and 27 in the *lys2Δ3′* allele (p = 0.09; panels A and B, respectively). Although the putative break sites occurred across the full length of both substrates, they were strongly skewed to the 3′ half of the *lys2Δ3′* allele (22/27 events; p = 0.002).

In addition to the spontaneous CO events that contained a hetDNA pattern consistent with initiation *via* a DSB, there were four events with the hetDNA pattern expected of initiation with a single-strand nick (Fig 7C). In these events, there was a single asymmetric hetDNA tract adjacent to the CO position (see Fig 1B). There also were two events with the hetDNA pattern predicted by recombination initiating with a single-strand gap (Fig 7D). In these COs, there was an asymmetric hetDNA tract extending from a symmetric tract at the presumptive CO point (see Fig 1C). Although consistent with initiation with an SSB, similar hetDNA patterns were seen among those COs induced by I-*Sce*I. Finally, the remaining 29 events had hetDNA patterns that did not fit those predicted by canonical recombination models, with most (21/29) having hetDNA in only one of the CO products. The profiles of these 29 events plus events containing no hetDNA are in S5 Fig.

As with DSB-initiated COs, we estimated the median length of hetDNA tracts in spontaneous CO events that contained hetDNA. For this analysis, the distances between the two most end-proximal transitions from heteroduplex to homoduplex DNA, or between donor and recipient sequence, were used. The median hetDNA length among the 77 events with hetDNA was 2060 bp (S1 Appendix). This is ~30% shorter than hetDNA associated with the repair of a centrally located DSB (2990 bp; p = 0.0026 by Mann Whitney U-test), which likely reflects the randomness of initiation sites.

### hetDNA tracts in ROS-associated CO events

**Fig 8:**
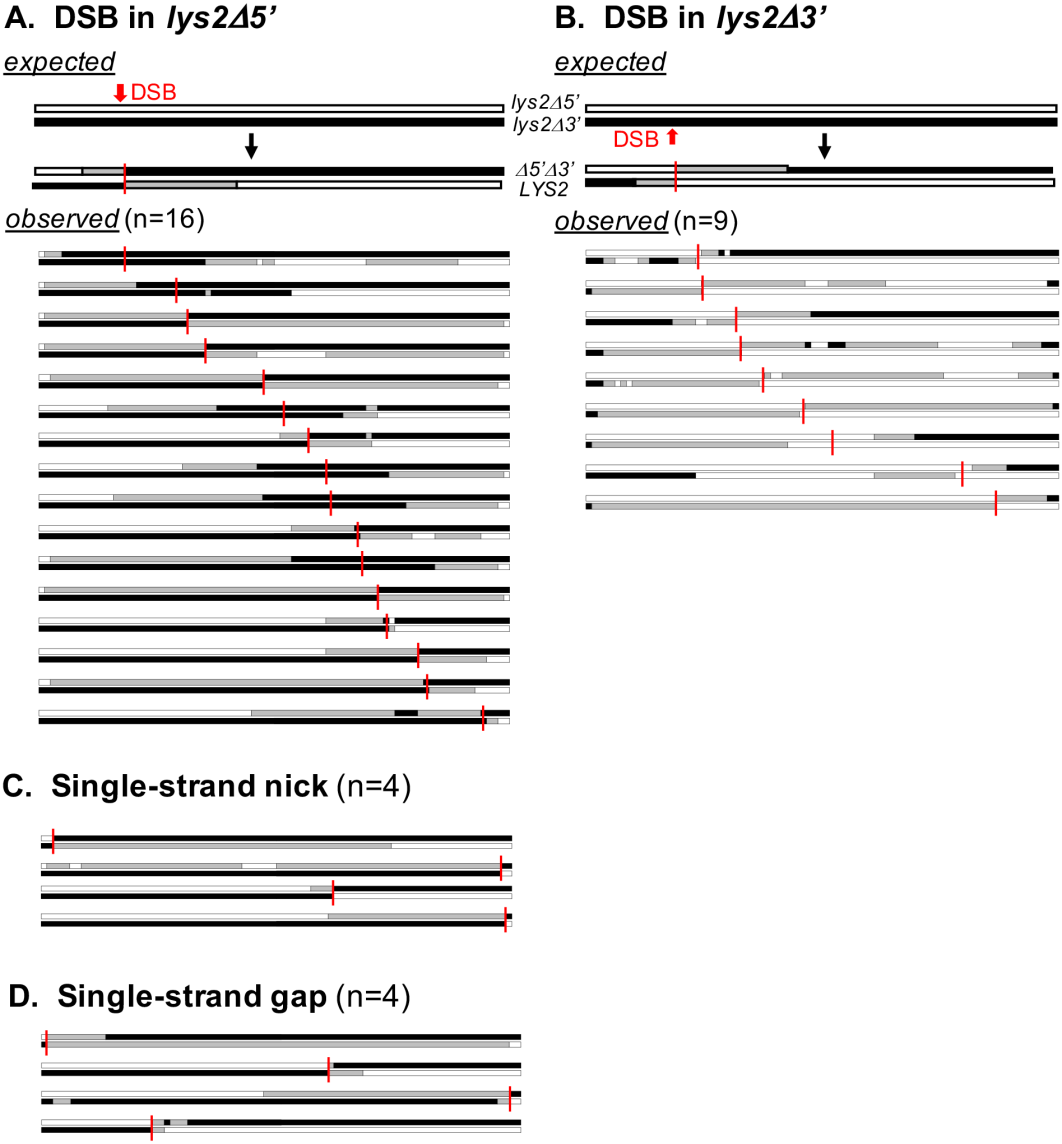
hetDNA profiles of spontaneous COs in a *tsa1Δ* background. White, black, and gray boxes represent *lys2Δ5*′, *lys2Δ3*′ and hetDNA, respectively. Potential initiation sites are marked with vertical red lines. (A) COs with a hetDNA pattern consistent with an initiating DSB in the *lys2Δ5*′ allele. (B) COs with a hetDNA pattern consistent with an initiating DSB in the *lys2Δ3*′ allele. (C) COs exhibiting a hetDNA pattern consistent with initiation by a single-strand nick. (D) COs containing a hetDNA pattern consistent with initiation by a single-strand gap. “n” is the number of events with the relevant hetDNA pattern.

*TSA1* encodes a key peroxiredoxin that scavenges hydrogen peroxide (H_2_O_2_; [27]), and its loss elevates genome instability [28,30]. There was a 14-fold increase in the rate of Lys^+^ colonies upon disruption of *TSA1*, indicating that >90% of COs reflected elevated ROS and the resultant DNA strand breaks. The hetDNA profiles of 119 COs isolated in the *tsa1Δ* background were examined and 57 of these contained hetDNA. Similar to the spontaneous hetDNA profile in wild type, most COs that contained hetDNA in both products had the asymmetric pattern associated with initiation *via* a DSB (25/33; Fig 8). Whereas most events in wild type initiated in the *lys2Δ3′* allele (27/42), most (16/25) in the *tsa1Δ* strain initiated in the *lys2Δ5′* allele (p = 0.042). As in the wild-type background, the presumptive site of the initiating DSB was distributed across the two substrates. There additionally were four events containing a hetDNA pattern consistent with a single-strand nick and four consistent with a single-strand gap as the primary initiating event. Finally, there were 62 and 24 events with only gene conversion tracts or uninterpretable hetDNA, respectively (S6 Fig). The median hetDNA length associated with COs in the *tsa1Δ* background was 3590 bp (S1 Appendix), which is 70% longer than that in the wild-type background (p = 0.005).

## Discussion

In this study, we report high-resolution mapping of hetDNA in CO events initiated with a defined DSB and in spontaneous events isolated in ROS scavenging-proficient and -deficient cells. Major advantages of the system include the dense placement of SNPs (1 SNP/50 bp) within the recombination substrates, the lack of a requirement for an associated gene conversion event and the ability to define hetDNA positions relative to a defined DSB. Only ~60% of the DSB-induced COs that contained hetDNA exhibited the two-sided, asymmetric hetDNA pattern predicted by the DSB repair model. Notably, all had the hetDNA tract upstream of the break in the *lys2Δ5′Δ3′* allele and the downstream of the break in the *LYS2* allele (Fig 3), which is the same biased hetDNA pattern we previously reported in a plasmid-chromosome assay [34]. In budding yeast, HJ resolvase candidates include the nucleases Mus81-Mms4 and Yen1, with only the latter exhibiting efficient activity on fully ligated junctions (reviewed by [40]). The resolution of fully ligated HJs is expected to produce both NCOs or COs, and the position of hetDNA within CO products is expected to be random. By contrast, nick/gap-directed HJ cleavage is expected to produce only CO products and also to generate the nonrandom hetDNA pattern observed here (see S3 Fig). It should be noted that this stereotypical hetDNA pattern is expected regardless of which end of the break initially invades the donor and whether there are one or two nicked HJs present. A preponderance of nicked relative to ligated HJs also explains why NCOs with the hetDNA pattern predicted by HJ cleavage were absent in a similar system [36].

The Rad1-Rad10 endonuclease was shown previously to be required for CO formation between ectopic, but not allelic, substrates and to act upstream of Mus81 and Yen1 [41]. Its activity was proposed to be necessary in two contexts: (1) removal an unpaired 3′ tail at the captured end of the DSB if DNA synthesis from the invading end extends beyond the shared homology and (2) cleavage a captured D-loop when 5′-end resection extends beyond the homology border and produces an adjacent gap. Among the COs analyzed, more than half had hetDNA that covered most of the homology, suggesting that extension of 3′ ends usually extends beyond the shared homology and creates over-replicated tails. The current system could be used to confirm a Rad1-Rad10 requirement for hetDNA tracts that extend to the homology border and to further delineate the *in vivo* roles of Mus81-Mms4 and Yen1 in HJ cleavage. In the absence of Mus81, for example, HJs may mature into a fully ligated form that is a substrate for Yen1. This in turn, would be expected to randomize the position of hetDNA in CO products.

In addition to the predominant two-sided, asymmetric hetDNA pattern, ~40% of hetDNA-containing CO products had the tract limited to only one side of the DSB. This class can be explained by a migrating D-loop model [42] in which further migration of the back end of the D-loop stops when a captured end is encountered (Fig 6A). A similar event in MMR-proficient cells would give rise to a single gene conversion tract located on only one side of the DSB. We previously reported frequent DSB-initiated allelic CO events with just a single gene conversion tract and attributed these to frequent restoration-type repair [43]. Because D-loop migration erases the hetDNA created by strand invasion, however, this may have contributed to the one-sided gene conversion tracts. In addition, D-loop migration past the break site was invoked to explain rare donor-recipient hetDNA transitions that were displaced from the DSB (Fig 6B). Finally, branch migration of the forward edge of the D-loop (or an HJ) after 3′-end extension ceases would convert asymmetric hetDNA into a break-distal, terminal symmetric tract (Fig 4B).

Although COs with one-sided hetDNA can be fit into the general framework of the canonical DSB repair model, a mechanism that terminates branch migration within 30–35 bp of engaged end, which is the position of next SNP beyond the terminal one removed by Pol δ, is required (Fig 6A). Interaction of the back end of the D-loop with the second end of the break creates a *bona fide* HJ, for example, and this is likely a much more static structure. An intriguing alternative is that D-loop processing frequently occurs prior to extension of the invading 3′ end, which precludes D-loop capture by the second end of the break (Fig 6C). Nicking of both donor strands of the D-loop by structure-specific endonucleases generates one CO product (after ligation of the nicked donor strand to the 3′ invading end) and releases the other arm of the donor chromosome. Analogous breakage of donor sequences has been detected during physical analysis of intermediates formed during multi-invasion rearrangements that reflect the simultaneously engagement of more than one donor by a single 3′ end [38] and during BIR [44]. What remains after the D-loop processing are the two arms of the reciprocal CO product, which are joined by synthesis-dependent strand annealing (SDSA). Depending on which broken end is extended and the template used, the resulting reciprocal CO products can contain either one- or two-sided hetDNA.

Prior mitotic [45] and meiotic [46] studies that mapped hetDNA reported interrupted/patchy patterns that were inconsistent with the simplest version of the DSB repair model. A distinct advantage of the CO-specific system used here is that the position of these complexities with respect to initiating break are known, allowing inferences to be made regarding gap expansion, template switching, HJ/branch migration, and/or MMR-independent mismatch correction. Approximately 40% of the DSB-associated COs with one-sided hetDNA tracts and 15% of those with two-sided hetDNA, for example, had a gene conversion tract that began precisely at the break and spanned multiple SNPs, consistent with frequent gap expansion. While such gap expansion was suggested as a possible source of gene conversion tracts in spontaneous COs [45] and was observed in physical analyses when mitotic repair was precluded [47], the known position of the DSB in the current system confirms that this indeed occurs. Gap expansion could reflect either removal of 3′ tails or, for a DSB that occurs in G1, run-off at a 5′ end resected prior to DNA replication. In prior analyses ~20% of NCO events generated by the SDSA pathway also experienced 3′ end loss [36], indicating that gap expansion is not unique to a specific HR subpathway. As previously reported for the SDSA pathway, there also was frequent removal of only a single SNP next to the DSB, which we assume similarly reflects the exonuclease activity of DNA polymerase δ [36]. Interestingly, the current analyses revealed that the efficiency of terminal-SNP removal reflects the corresponding mismatch created by strand invasion/annealing. Although we previously argued that single-SNP removal is likely due to exonuclease activity of Pol δ on free 3′ tails created if pairing with the donor initiates internally, the current results are more consistent with proofreading activity.

Invasion of a donor repair template by a 3′ tail is expected to produce continuous hetDNA, but we often observed embedded gene conversion or restoration events. These events presumably reflect a MMR-independent repair system. Following donor engagement, the gaps created by 5′-end resection are filled using the donor as a template and are expected to be free of hetDNA (Fig 1). We frequently observed hetDNA embedded in the donor portion of CO products, however, and this can be explained by a temporary template switch to the sister of the broken molecule (Fig 4C).

Studies of the mitotic repair of large gaps in Drosophila were the first to suggest that multiple cycles of donor invasion are often required [48]. In yeast, template switching was initially described in the context of the break-induced replication (BIR) pathway [49], which requires extensive DNA synthesis to repair a break that has homology at only one end (reviewed by [50]). More recently, however, template switching between chromosomes was reported in up to 0.3% of DSB-initiated gene conversion events in a selective system [51]. In the current system, where there was no selection for template switching and less than 2 kb of DNA synthesis was required, the level approached an astonishing 20%. BIR depends on the Pol32 subunit of Pol δ [52], and we have shown that ectopic COs (but not NCOs) are similarly very dependent on Pol32 [36]. The high frequency of template switching during mitotic recombination as well as the strong dependence of ectopic CO events on Pol32 suggests there may be little that distinguishes the associated DNA synthesis from that typically associated with BIR.

**Fig 9:**
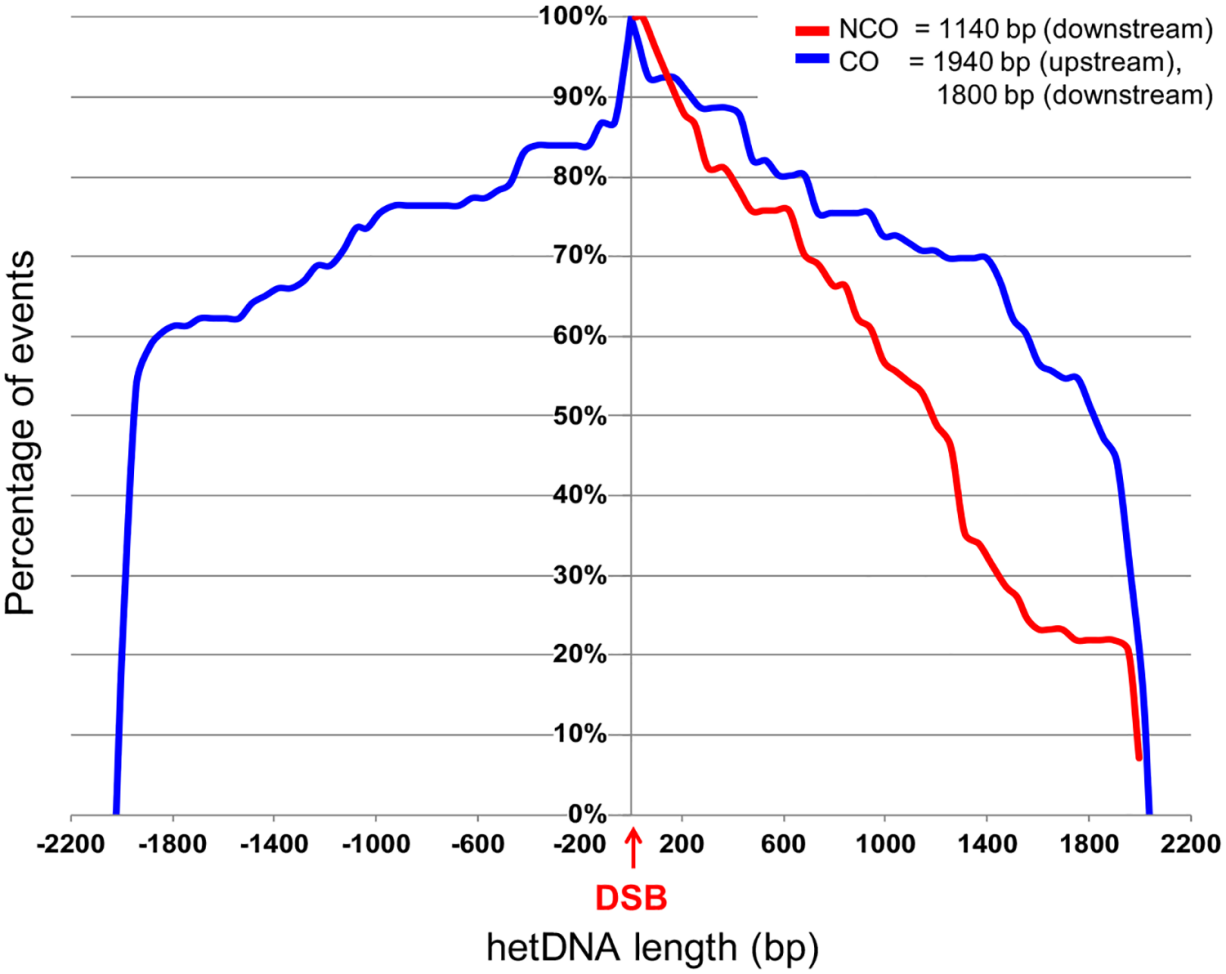
Distribution of hetDNA associated with COs and SDSA-type NCOs. The distances of SNPs from the DSB site (position 0; negative numbers are for upstream distances) are shown on the x-axis and the percentages of repair events with hetDNA that extends a given distance are on the y-axis. CO-associated hetDNA length distributions on each side of the DSB (blue lines) are from the current data (see S1 Appendix). The NCO-associated hetDNA length distribution (red line) on the downstream side of the DSB was previously reported [36]. Median hetDNA lengths are indicated.

Ectopic substrates similar to the CO-specific substrates used here were recently used to analyze the one-sided hetDNA characteristic of SDSA-generated NCOs events [36]. Fig 9 presents the length distribution of hetDNA in these SDSA events along with that of downstream hetDNA in the COs examined here. Though the length of homology on each side of the induced DSB was similar in the two systems (~2 kb), the median length of CO-associated hetDNA was ~1.9 kb, while that associated with SDSA events was only ~1.1 kb (p = 0.0002). The significant hetDNA length difference between COs and NCOs in similar ectopic systems, but not in allelic systems [43,45], is consistent with our previous suggestion that the second-end engagement, which is a prerequisite for reciprocal CO formation in the canonical DSB repair pathway, requires more extensive homology length than does SDSA [36,53]. We suggested that the D-loop size and stability is directly related to the length of hetDNA that forms during the initial strand invasion, which may be limited by substrate size in ectopic assays. This limitation will favor a NCO outcome.

Gene conversion tracts associated with DSB-induced and spontaneous NCOs in a plasmid-chromosome assay with limited homology were previously examined, and spontaneous tracts were ~40% longer [54]. The current work compared CO-associated hetDNA profiles in a chromosomal context where substrates shared more extensive homology and MMR was inactivated, which allowed the transition of hetDNA from one product to the other to be monitored. In contrast to the previous analysis of gene conversion tracts, the hetDNA associated with spontaneous CO events was less extensive than in DSB-initiated events. Furthermore, more than half of the hetDNA associated spontaneous COs recovered from WT (55%) and *tsa1Δ* (67%) cells had the two-sided asymmetric hetDNA tracts indicative of initiation by a DSB, which was similar to the proportion among DSB-induced COs. Although the hetDNA patterns predicted for a nick or gap-initiated CO also were observed among spontaneous COs recovered from WT (8%) and *tsa1Δ* (17%) strains, similar patterns were frequent among DSB-induced COs. Based on associated hetDNA patterns, we conclude that nicks likely trigger canonical HR only following conversion to a DSB. A similar conclusion regarding SSB conversion to a DSB was reached in earlier yeast studies that used a phage nickase to initiate recombination [12,13]. A result that remains unexplained is the existence of yeast *rad52* mutants that are sensitive to ionizing radiation and defective in the repair of induced DSBs, but have normal levels of spontaneous recombination. This led to the conclusion that DSBs are unlikely to be the primary initiating lesion of spontaneous events [20]. In contrast to the CO-specific assay used here, however, assays that detected primarily gene conversion events were used. It is possible that spontaneous gene conversions primarily reflect a gap-filling reaction while most COs are initiated by DSBs. Finally, we note that the distribution of spontaneous recombination products in mammalian cells differed from that initiated by I-*Sce*I cleavage [55].

COs from an ROS scavenging-deficient (*tsa1Δ*) strain also had spontaneous hetDNA patterns consistent with initiation primarily by DSBs, but there were differences relative to WT in terms of initiation sites and median hetDNA length. Whereas spontaneous breaks in the WT background were biased toward the 3′ end of one allele, initiation sites were random in the *tsa1Δ* mutant. With elevated ROS levels, DSB sites likely reflect clustered damaged bases, abasic sites and/or SSBs [56], which would be expected to be randomly located throughout the recombination substrates. By contrast, DSBs in wild-type cells may be primarily generated during DNA transactions such as DNA replication or transcription, resulting in biased initiation sites. A second notable difference between COs in *TSA1* and *tsa1Δ* cells was the length of associated hetDNA, which was significantly longer in the *tsa1Δ* mutant than in WT, which could reflect the relatively random positions of initiating DSBs. In WT, more than half of the DSBs appeared to initiate close to the 3′ end of one recombination substrate, which limits homology on one side of the break. Alternatively, the longer hetDNA tracts in *tsa1Δ* cells could reflect a saturation of mismatch binding capacity caused by elevated ROS throughout the genome. This would relieve the inhibitory effect of mismatch binding on HR, which triggers heteroduplex rejection and likely limits hetDNA formation (reviewed in [57]).

In summary, molecular analyses of hetDNA tracts associated with the repair of a defined DSB has revealed a high degree of complexity that is not predicted by the canonical version of DSB repair model. These complexities are consistent with frequent gap expansion, D-loop (and possibly HJ) mobility, template-switching during 3′ end extension and MMR-independent hetDNA repair. The DSB repair model predicts a hetDNA tract on each side of the initiating break, but only 60% of the CO products that contained hetDNA had this pattern. Furthermore, the positions of these tracts in the products were not random, suggesting that HJ resolution is nick (or gap) directed. The frequent occurrence of hetDNA on only one side of the initiating break could reflect either D-loop migration that halts at the break site when the second end is captured, or D-loop processing that occurs prior to extension of the invading end. Finally, the hetDNA profiles associated with spontaneous COs were similar to those in DSB-initiated events, providing molecular evidence that DSBs are the primary physiological initiator of spontaneous recombination. Because HR mechanisms are highly conserved through evolution, results in the yeast system should be broadly relevant to higher eukaryotes.

## Materials and methods

### Yeast strain constructions

A complete strain list is presented in S1 Table. All haploid strains were derived from MC42-2d (= SJR3659) and HLK1042-1c (= SJR3782), both of which are *RAD5 CAN1* derivatives of W303 *(rad5-535 leu2-3*,*112 his3-11*,*15 ura3-1 ade2-1 trp1-1 can1-100*; [58]) constructed by transformation [59]. Two types of strains were used in this study. The first was used to detect DSB-induced CO events and contained three components: an I-*Sce*I-cleavable, 5′-truncated *lys2* allele (*kanMX-lys2Δ5*′::*ade2*,*I-SceI*) at the endogenous *LYS2* locus on chromosome II; a 3′-truncated *lys2* gene containing a mutated, non-cleavable I-*Sce*I site (*can1*::*lys2Δ3*′*-98%*,*ade2,I-SceInc*) located at the *CAN1* locus on chromosome V; and a galactose-inducible I-*Sce*I gene replacing the endogenous *HIS3* gene (*his3Δ*::*hph-pGAL-*I*-Sce*I) on chromosome XV. The second type of strain lacked the galactose-inducible I-*Sce*I gene and contained only the 5′- and 3′-truncated *lys2* alleles, allowing detection of spontaneous CO events.

The *kanMX-lys2Δ5*′::*ade2*,*I-SceI* allele (hereafter referred to as *lys2Δ5*′) was constructed by modifying the intact *lys2* allele of strain SJR3714 [36]. In SJR3714, an I-*Sce*I recognition site was inserted ~ 400 bp from the start codon of *LYS2*. We first increased the homology between recombination substrates so that it extended ~2 kb upstream of the I-*Sce*I cut site. This was done by inserting a segment containing nt 7–1710 of the *ADE2* gene (amplified from pRS402; [60] immediately upstream of the I-*Sce*I cut site using the *delitto perfetto* method [61]. The endogenous *LYS2* promoter and the first 100 bp of the *LYS2* reading frame were then replaced with the kanamycin-resistance marker from pFA6a-kanMX6 [62], resulting in haploid strain SJR4533.

The 4.5 kb *lys2Δ3*′*-98%*,*ade2*,*I-SceInc* allele (hereafter referred to as *lys2Δ3*′) was synthesized by Life Technologies and contained 82 silent SNPs at approximately 50-bp intervals (see S1 Fig). The synthesized *lys2Δ3*′ allele was truncated at nt position 2428 of the *LYS2* open reading frame and included the endogenous *LYS2* promoter as well as the *ADE2* insertion and a noncleavable I-*Sce*I cut site (*I-SceInc*) at the same position as in the *lys2Δ5*′ allele. A *Pvu*II-digested fragment containing the *lys2Δ3*′ allele was cloned into *Msc*I-digested pSR797 to generate pSR1132, which has the *lys2Δ3*′ allele inserted in *CAN1* sequence. pSR797 contains nt 20–1141 of the *CAN1* open reading frame [36]. Because the *lys2Δ5*′ allele contained most of the *ADE2* sequence, intermediate strain SJR4277 was created by replacing the endogenous *ADE2* gene of SJR3782 with a *loxP-TRP1-loxP* cassette from plasmid pSR954 [kanamycin-resistance marker of pUG6 (Guldener et al., 1996) replaced with *TRP1* from pFA6-TRP1 [62]]. A*fl*II/X*cm*I-digested pSR1132 was then used to transform SJR4277, yielding SJR4468. Strain SJR4304, a SJR3714 derivative containing a galactose-inducible I-*Sce*I gene (amplified from pGSHU; [63] and an *MLH1* deletion (*mlh1Δ*::*loxP-TRP1-loxP*), was then mated with SJR4468 to generate strain SJR4469 that contained the *lys2Δ3*′ allele, galactose-regulated I-*Sce*I and deletions of *ADE2* and *MLH1*.

Haploid strains with all three components for the CO-specific systems (SJR4608) or with only the *lys2Δ5*′ and *lys2Δ3*′ alleles (SJR4534, SJR4536, and SJR4606) were created by mating SJR4469 and SJR4533. A *TSA1* deletion was created by one-step replacement of one copy of *TSA1* in diploid strain SJR4614 with a *tsa1Δ*::*loxP-URA3Kl-loxP* PCR fragment (from pUG72; [64], generating SJR4627. SJR4614, which was created by mating SJR4536 and SJR4606, contained the *lys2Δ5*′ and *lys2Δ3*′ alleles and was heterozygous at the *MLH1* locus. The diploid SJR4627 was sporulated and dissected to obtain the *tsa1Δ mlh1Δ* haploid strains SJR4672 (*MLH1*) and SJR4674 (*mlh1Δ*) for detection of spontaneous COs.

### Media and growth conditions

All growth was at 30°C. Except for experiments involving galactose induction, cells were grown nonselectively in YEPD (1% Bacto-yeast extract, 2% peptone, 2% dextrose; 1.5% agar for plates) supplemented with 500 μg/ml adenine hemisulfate. For galactose induction of I-*Sce*I, cells were pre-grown in YEPR (1% Bacto-yeast extract, 2% peptone, 2% raffinose) prior to addition of an appropriate amount of galactose. Unless otherwise noted, selective growth was on synthetic complete (SC) medium lacking lysine and containing 2% dextrose as a carbon source.

### I-*Sce*I induction and recovery of DSB-induced COs

Prior to galactose induction of I-*Sce*I, individual YEPR liquid cultures were inoculated with 2-day old yeast colonies (SJR4608) and grown overnight. Following overnight expansion, individual cultures were diluted to an optical density (O.D.) of 0.2 with fresh YEPR liquid medium and split into two parallel cultures. To ensure experiments were performed in exponentially growing cultures, the diluted cultures were re-grown to an O.D. of 0.8–1 before galactose addition (final concentration of 0.1%) to one of each culture pair. As previously determined, galactose induction with 0.1% galactose for 45 minutes generally resulted in cleavage of only one sister chromatid in exponentially growing cells [36]. Following exposure to galactose, cells from each culture pair were diluted appropriately and plated on dextrose-containing SC-lys plates. After three days of growth, randomly selected Lys^+^ colonies were directly inoculated into SC-lys medium in 96-well microtiter plates. Genomic DNA was extracted after ~24-hour growth and used for PCR amplification for hetDNA analysis (see below). The hetDNA profiles obtained were based on 166 independent colonies from two independent inductions.

### Spontaneous CO rates and recovery of spontaneous COs

To compare spontaneous CO rates between *TSA1* (SJR4606) and *tsa1Δ* (SJR4672) strains, individual YEPD cultures were inoculated with approximately 5000 cells from independent colonies. Cultures were grown at 30°C until saturated. Appropriate dilutions of saturated cultures were plated on YEPD to determine the total number of viable cells and on SC-lys with dextrose to determine the number of Lys^+^ colonies. Spontaneous CO rates were calculated using the method of the median [65] and the associated 95% confidence intervals were determined as previously described [66]. Spontaneous CO rates in WT and *tsa1Δ* were determined based on three independent experiments, with 12 independent cultures per experiment.

Spontaneous COs from *TSA1* (SJR4534) and *tsa1Δ* (SJR4674) strains were collected for hetDNA analyses in the absence of *MLH1*. hetDNA profiles were obtained from Lys^+^ colonies from two independent experiments. For each experiment, YEPD cultures inoculated with independent colonies were expanded overnight to saturation. Because spontaneous lesions are infrequent and arise randomly during normal growth, cells were plated as follows to ensure recovery of independent spontaneous events and preservation of the hetDNA footprint within Lys^+^ colonies. First, cells were diluted and spotted on a nonselective agar plate using a prong with a hundred flat-tipped pins such that approximately 10^5^ cells/pin were transferred. Each spot corresponding to a mini culture, allowing parallel, high-throughput identification of spontaneous COs. Only a small number of cells was initially transferred to nonselective medium to prevent seeding of pre-existing HR events. The absence of Lys^+^ recombinants was confirmed by simultaneous spotting of directly onto selective medium. Cells on the nonselective medium were grown overnight to allow accumulation of spontaneous HR events and then were replica-plated to selective SC-lys medium. After replica plating, spontaneous CO events occurring during non-selective growth were identified as Lys^+^ colonies.

### hetDNA analysis

hetDNA profiles were determined using SMRT sequencing as previously described [36] with some modifications. To obtain a complete hetDNA profile for each event, each CO product (~4 kb) was amplified as a single fragment from genomic DNA extracted from unpurified Lys^+^ colonies. High fidelity Phusion polymerase (New England Biolabs) was used in conjunction with primers in which unique 16-nt PacBio barcodes were anchored to “universal” forward and reverse primer pairs specific for the *LYS2* and *lys2Δ5*′*Δ3*′ products. The list of PacBio barcodes is available at https://github.com/PacificBiosciences/Bioinformatics-Training/blob/master/barcoding/pacbio_384_barcodes.fasta. Each barcoded primer pair used to amplify the *LYS2* product was linked to forward (5′ CTTTTTACGCCCACAACAAGAACC) and reverse (5′ GCTTGGGAGTTGGGAATTGAAGTT) universal sequences. The forward and reverse universal sequences for amplification of the *lys2Δ5*′*Δ3*′ product were 5′-ACGAGCTCGAATTCATCGATGATA and 5′-TCACTTTTGCCCTGGAACTTAGTG, respectively. ImageJ (https://imagej.nih.gov/ij/) was used to determine amplicon concentrations and equivalent amounts of individual barcoded amplicons were pooled for SMRT library construction and sequencing. Barcoded libraries were sequenced using at Duke Center for Genomic and Computational Biology using the PacBio RSII system.

Following sequencing, barcoded recombinants were sorted using an in-house pipeline previously described (Guo et al., 2015). The reference sequences of the *lys2Δ5*′ and *lys2Δ3*′ substrates used for circular consensus sequence (CCS) read alignment and variant calling are in S1 Fig. Because recombinants were isolated in *mlh1Δ* strains, hetDNA was detected as region of SNPs contributed by both of the parent alleles (*lys2Δ5*′ and *lys2Δ3*′). For each recombinant, CCS species were sorted by their SNP profiles. Recombinants with more than two major CCS species were regarded as spurious and were eliminated from hetDNA analyses. The unprocessed CCS reads in a FASTA file format and the unique barcodes used are available at http://dx.doi.org/10.17632/kp6bzpg6p5.1.

### Statistical analysis

hetDNA distributions were compared by two-sided Fisher exact or chi-square tests. Distributions of hetDNA lengths were compared using the Mann-Whitney U test.

## Supporting information

S1 Table Yeast strains. (PDF)

S1 Fig SNPs in CO-specific recombination substrates. The region of the *lys2Δ5*′ allele that is homologous to the *lys2Δ3*′ allele is shown. The segment introduced from the *ADE2* gene is highlighted in blue; the introduced I-*Sce*I site is highlighted yellow, with the region flanked by enzyme-generated nicks underlined. In the *lys2Δ3*′ allele, an additional 6 nt (CCATAA) was added after the underlined region in order to maintain the reading frame while preventing I-*Sce*I cleavage. SNPs introduced into the donor allele are above the sequence and are highlighted gray.
(PDF)

S2 Fig DSB-associated two-sided hetDNA. The *lys2Δ5*′*Δ3*′ and *LYS2* alleles for each CO event in Fig 3 are shown stacked rather than side-by-side to allow visualization of hetDNA and gene conversion transitions. The *lys2Δ5*′*Δ3*′ is at the top of each pair of products and the *LYS2* allele at the bottom. Events are aligned vertically based on the upstream hetDNA length so that their order is as in Fig 3. The vertical red line indicates the position of the initiating DSB.
(PDF)

S3 Fig hetDNA position following nick-directed or random HJ cleavage. Red and black lines represent the *lys2Δ5′* and *lys2Δ3′* alleles, respectively. Arrowheads mark the 3′ ends of DNA strands and yellow boxes the hetDNA. Black and gray triangles indicate sites of HJ cleavage. (A) Nick-directed HJ cleavage (cleavage of the strand with the same polarity as the nicked strand; black triangles) occurs before HJs are fully ligated. This generates CO products with hetDNA always upstream and downstream of the DSB in the *lys2Δ5’Δ3’* and *LYS2* alleles, respectively. The same pattern is produced regardless of which broken end invades the repair template. (B) Random cleavage occurs when the HJs are fully ligated. Alternative cleavage sites that produce CO products are represented by the black and gray triangles. Cleavage at the positions of the black triangles produces the same hetDNA pattern as nick-directed cleavage, while cleavage at the positions of the gray triangles reverses the hetDNA positions relative to the DSB.
(PDF)

S4 Fig DSB-associated one-sided hetDNA. The *lys2Δ5*′*Δ3*′ and *LYS2* alleles for each CO event in Fig 5 are shown stacked rather than side-by-side, and are in the same order as in Fig 5. The *lys2Δ5*′*Δ3*′ is at the top of each pair of products and the *LYS2* allele at the bottom. The vertical red line indicates the position of the initiating DSB. (A) hetDNA on either the left or right side of the DSB. (B) The transition of asymmetric hetDNA from one product to the other is displaced from the DSB and is indicated by the blue vertical line. “n” is the number of events with the relevant hetDNA pattern.
(PDF)

S5 Fig DSB-initiated COs with hetDNA confined to the donor allele or no hetDNA. CO products for each event are stacked; the *lys2Δ5′Δ3′* allele is on the top and the *LYS2* allele on the bottom of each pair. (A) hetDNA was confined to the donor allele. (B) hetDNA was absent in CO products, but gene conversion tracts were present. The vertical red line indicates the position of the initiating DSB. “n” is the number of events with the relevant hetDNA pattern.
(PDF)

S6 Fig Spontaneous COs lacking het DNA or with indeterminate hetDNA. All events were isolated in a wild-type (*TSA1*) background. CO products for each event are stacked; the *lys2Δ5′Δ3′* allele is on the top and the *LYS2* allele on the bottom of each pair. (A) CO products with no detectable hetDNA. (B) CO events with hetDNA confined to only one allele. (C) CO events with hetDNA in both alleles, but with a “patchy” pattern that did not match that predicted for a DSB/nick/gap-induced COs (see Fig 1).
(PDF)

S7 Fig Spontaneous COs lacking het DNA or with indeterminate hetDNA. All events were isolated in a *tsa1Δ* background. CO products for each event are stacked; the *lys2Δ5′Δ3′* allele is on the top and the *LYS2* allele on the bottom of each pair. (A) CO products with no detectable hetDNA. (B) CO events with hetDNA confined to only one allele. (C) CO events with hetDNA in both alleles, but with a “patchy” pattern that did not match that predicted for a DSB/nick/gap-induced CO (see Fig 1).
(PDF)

S1 Appendix hetDNA lengths for DSB-induced, spontaneous *TSA1*, and spontaneous *tsa1Δ*. (XLSX)
