# Supplementary material for: DNA strand-exchange patterns associated with double-strand break-induced and spontaneous mitotic crossovers in Saccharomyces cerevisiae
Source: PLoS Genet. 2018 Mar 26;14(3):e1007302. doi: 10.1371/journal.pgen.1007302 (PMC5886692; doi:10.1371/journal.pgen.1007302)
Supplement: S1 Table — (PDF) [file pgen.1007302.s001.pdf]

Table S1. Yeast strains

Strain	Relevant genotype[a]	Comments/reference
SJR3659	*MATa RAD5 leu2-3,112 his3-11,15 ura3-1 trp1-1 ade2-1 CAN1*	Strain MC42-2d
SJR3714	*MATa lys2::I-SceI*	Lab strain
SJR3782	*MATα RAD5 leu2-3,112 his3-11,15 ura3-1 trp1-1 ade2-1 CAN1 hom3-10*	Strain HLK1042-1c
SJR4015	*MATa lys2*::CORE	SJR3714 with CORE cassette inserted into *LYS2*
SJR4277	*MATα ade2Δ::loxP-TRP1-loxP*	SJR3782 *ade2Δ*
SJR4304	*MATa his3Δ::pGAL-I-SceI mlh1Δ::loxP-TRP1-loxP hisG-lys2::I-SceI can1::lys2Δ3′-98%,I-SceInc-URA3-hisG*	SJR3714 *mlh1Δ* with *pGAL-I-SceI* and CO-NCO substrates
SJR4468	*MATα ade2Δ::loxP-TRP1-loxP can1::lys2Δ3′-98%,ade2,I-SceInc*	SJR4277 transformed with *Afl*II-*Xcm*I digested pSR1132
SJR4469	*MATα HOM3 ade2Δ::loxP-TRP1-loxP mlh1Δ::loxP-TRP1-loxP his3Δ::pGAL-I-SceI can1::lys2Δ3'-98%,ade2,I-SceInc HOM3*	Spore from SJR4304 x SJR4468
SJR4533	*MATa kanMX-lys2Δ5′::ade2,I-SceI*	Derivative of SJR4015 with the *lys2Δ5′* allele
SJR4534	*MATα ade2Δ::loxP-TRP1-loxP mlh1Δ::loxP-TRP1-loxP kanMX-lys2Δ5′::ade2,I-SceI can1::lys2Δ3′-98%,ade2,I-SceInc*	Spore from SJR4533 x SJR4469
SJR4536	*MATa ade2Δ::loxP-TRP1-loxP mlh1Δ::loxP-TRP1-loxP kanMX-lys2Δ5′::ade2,I-SceI can1::lys2Δ3′-98%,ade2,I-SceInc*	Spore from SJR4533 x SJR4469
SJR4606	*MATα ade2Δ::loxP-TRP1-loxP kanMX-lys2Δ5′::ade2,I-SceI can1::lys2Δ3′-98%,ade2,I-SceInc*	Spore from SJR4533 x SJR4469
SJR4608	*MATα ade2Δ::loxP-TRP1-loxP mlh1Δ::loxP-TRP1-loxP his3Δ::pGAL-I-SceI kanMX-lys2Δ5′::ade2,I-SceI can1::lys2Δ3′-98%,ade2,I-SceInc*	Spore from SJR4533 x SJR4469
SJR4614	*MATa/MATα ade2Δ::loxP-TRP1-loxP/ade2Δ::loxP-TRP1-loxP MLH1/mlh1Δ::loxP-TRP1-loxP kanMX-lys2Δ5′::ade2,I-SceI/kanMX-lys2Δ5′::ade2,I-SceI can1::lys2Δ3′-98%,ade2,I-SceInc/can1::lys2Δ3′-98%,ade2,I-SceInc*	Diploid SJR4536 x SJR4606
SJR4627	*MATa/MATα ade2Δ::loxP-TRP1-loxP/ade2Δ::loxP-TRP1-loxP MLH1/mlh1Δ::loxP-TRP1-loxP kanMX-lys2Δ5′::ade2,I-SceI/kanMX-lys2Δ5′::ade2,I-SceI can1::lys2Δ3′-98%,ade2,I-SceInc/can1::lys2Δ3′-98%,ade2,I-SceInc TSA1/tsa1Δ::loxP-URA3kl-loxP*	Diploid SJR4614 with heterozygous *TSA1* deletion
SJR4672	*MATα ade2Δ::loxP-TRP1-loxP MLH1 kanMX-lys2Δ5′::ade2,I-SceI can1::lys2Δ3′-98%,ade2,I-SceInc tsa1Δ::loxP-URA3kl-loxP*	Spore from SJR4627

SJR4674	*MATα ade2Δ::loxP-TRP1-loxP mlh1Δ::loxP-TRP1-loxP kanMX-lys2Δ5′::ade2,I-SceI can1::lys2Δ3′-98%,ade2,I-SceInc tsa1Δ::loxP-URA3kl-loxP*	Spore from SJR4627

[a] All strains are isogenic with W303 derivatives SJR3659 (MC42-2d) and SJR3782 (HLK1042-1c) except for changes introduced by transformation or mating. Only differences from the progenitor strains SJR3782 and SJR3659 are given.
