# Supplementary material for: DNA strand-exchange patterns associated with double-strand break-induced and spontaneous mitotic crossovers in Saccharomyces cerevisiae
Source: PLoS Genet. 2018 Mar 26;14(3):e1007302. doi: 10.1371/journal.pgen.1007302 (PMC5886692; doi:10.1371/journal.pgen.1007302)
Supplement: S1 Fig — The region of the lys2Δ5′ allele that is homologous to the lys2Δ3′ allele is shown. The segment introduced from the ADE2 gene is highlighted in blue; the introduced I-SceI site is highlighted yellow, with the region flanked by enzyme-generated nicks underlined. In the lys2Δ3′ allele, an additional 6 nt (CCATAA) was added after the underlined region in order to maintain the reading frame while preventing I-SceI cleavage. SNPs introduced into the donor allele are above the sequence and are highlighted gray. (PDF) [file pgen.1007302.s002.pdf]

G C T
AAGCTACATATTCGTTACAGCTACCTCAGCTCGATGTGCCTCATGATAGTTTTTCTAACAAATACGCTGTCGCTTTGAGTGTATGGGCTGCATTGATATATAGAGTAACCGGTGACGATGATATTGTTCTTTATATTGCG

T T C
AATAACAAAATCTTAAGATTCAATATTCAACCAACGTGGTCATTTAATGAGCTGTATTCTACAATTAACAATGAGTTGAACAAGCTCAATTCTATTGAGGCCAATTTTTCCTTTGACGAGCTAGCTGAAAAAATTCAAAGTT

A C C
GCCAAGATCTGGAAAGGACCCCTTCTAGAACAGTTGGTATATTAGGAGGGGGACAATTGGGACGTATGATTGTTGAGGCAGCAAACAGGCTCAACATTAAGACGGTAATACTAGATGCTGAAAATTCTCCTGCCAAAC

C T G
AAATAAGCAACTCCAATGACCACGTTAATGGCTCCTTTTCCAATCCTCTTGATATCGAAAAACTAGCTGAAAAATGTGATGTGCTAACGATTGAGATTGAGCATGTTGATGTTCCTACACTAAAGAATCTTCAAGTAAAAC

T T
ATCCCAAATTAAAAATTTACCCTTCTCCAGAAACAATCAGATTGATACAAGACAAATATATTCAAAAAGAGCATTTAATCAAAAATGGTATAGCAGTTACCCAAAGTGTTCCTGTGGAACAAGCCAGTGAGACGTCCCTAT

T C A
TGAATGTTGGAAGAGATTTGGGTTTTCCATTCGTCTTGAAGTCGAGGACTTTGGCATACGATGGAAGAGGTAACTTCGTTGTAAAGAATAAGGAAATGATTCCGGAAGCTTTGGAAGTACTGAAGGATCGTCCTTTGTA

C C C
CGCCGAAAAATGGGCACCATTTACTAAAGAATTAGCAGTCATGATTGTGAGATCTGTTAACGGTTTAGTGTTTTCTTACCCAATTGTAGAGACTATCCACAAGGACAATATTTGTGACTTATGTTATGCGCCTGCTAGAG

A G A
TTCCGGACTCCGTTCAACTTAAGGCGAAGTTGTTGGCAGAAAATGCAATCAAATCTTTTCCCGGTTGTGGTATATTTGGTGTGGAAATGTTCTATTTAGAAACAGGGGAATTGCTTATTAACGAAATTGCCCCAAGGCCT

C C
CACAACTCTGGACATTATACCATTGATGCTTGCGTCACTTCTCAATTTGAAGCTCATTTGAGATCAATATTGGATTTGCCAATGCCAAAGAATTTCACATCTTTCTCCACCATTACAACGAACGCCATTATGCTAAATGTT

T C G
CTTGGAGACAAACATACAAAAGATAAAGAGCTAGAAACTTGCGAAAGAGCATTGGCGACTCCAGGTTCCTCAGTGTACTTATATGGAAAAGAGTCTAGACCTAACAGAAAAGTAGGTCACATAAATATTATTGCCTCCA

A C T
GTATGGCGGAATGTGAACAAAGGCTGAACTACATTACAGGTAGAACTGATATTCCAATCAAAATCTCTGTCGCTCAAAAGTTGGACTTGGAAGCAATGGTCAAACCATTGGTTGGAATCATCATGGGATCAGACTCTGA

G C G
CTTGCCGGTAATGTCTGCCGCATGTGCGGTTTTAAAAGATTTTGGCGTTCCATTTGAAGTGACAATAGTCTCTGCTCATAGAACTCCACATAGGATGTCAGCATATGCTATTTCCGCAAGCAAGCGTGGAATTAAAACA

C C
ATTATCGCTGGAGCTGGTGGGGCTGCTCACTTGCCAGGTATGGTGGCTGCAATGACACCACTTCCTGTCATCGGTGTGCCCGTAAAAGGTTCTTGTCTAGATGGAGTAGATTCTTTACATTCAATTGTGCAAATGCCT

C T C
AGAGGTGTTCCAGTAGCTACCGTCGCTATTAATAATAGTACGAACGCTGCGCTGTTGGCTGTCAGACTGCTTGGCGCTTATGATTCAAGTTATACAACGAAAATGGAACAGTTTTTATTAAAGCAAGAAGAAGAAGTTC

C T A G
TTGTCAAAGCACAAAAGTTAGAAACTGTCGGTTACGAAGCTTATCTAGAAAACCCTAGGGATAACAGGGTAATATTGTTCCGTTTGGCCTTTTTGGAAAACCAAGATTTCAAATTAGACGAGTTCAAGCATCATTTAGTG

C G C
GACTTTGCTTTGAATTTGGATACCAGTAATAATGCGCATGTTTTGAACTTAATTTATAACAGCTTACTGTATTCGAATGAAAGAGTAACCATTGTTGCGGACCAATTTACTCAATATTTGACTGCTGCGCTAAGCGATCCA

A T G
TCCAATTGCATAACTAAAATCTCTCTGATCACCGCATCATCCAAGGATAGTTTACCTGATCCAACTAAGAACTTGGGCTGGTGCGATTTCGTGGGGTGTATTCACGACATTTTCCAGGACAATGCTGAAGCCTTCCCAG

T C
AGAGAACCTGTGTTGTGGAGACTCCAACACTAAATTCCGACAAGTCCCGTTCTTTCACTTATCGCGACATCAACCGCACTTCTAACATAGTTGCCCATTATTTGATTAAAACAGGTATCAAAAGAGGTGATGTAGTGATG

T G G
ATCTATTCTTCTAGGGGTGTGGATTTGATGGTATGTGTGATGGGTGTCTTGAAAGCCGGCGCAACCTTTTCAGTTATCGACCCTGCATATCCCCCAGCCAGACAAACCATTTACTTAGGTGTTGCTAAACCACGTGGGT

C A G
TGATTGTTATTAGAGCTGCTGGACAATTGGATCAACTAGTAGAAGATTACATCAATGATGAATTGGAGATTGTTTCAAGAATCAATTCCATCGCTATTCAAGAAAATGGTACCATTGAAGGTGGCAAATTGGACAATGGC

T T C
GAGGATGTTTTGGCTCCATATGATCACTACAAAGACACCAGAACAGGTGTTGTAGTTGGACCAGATTCCAACCCAACCCTATCTTTCACATCTGGTTCCGAAGGTATTCCTAAGGGTGTTCTTGGTAGACATTTTTCCTT

C G
GGCTTATTATTTCAATTGGATGTCCAAAAGGTTCAACTTAACAGAAAATGATAAATTCACAATGCTGAGCGGTATTGCACATGATCCAATTCAAAGAGATATGTTTACACCATTATTTTTAGGTGCCCAATTGTATGTCCC

G C T
TACTCAAGATGATATTGGTACACCGGGCCGTTTAGCGGAATGGATGAGTAAGTATGGTTGCACAGTTACCCATTTAACACCTGCCATGGGTCAATTACTTACTGCCCAAGCTACTACACCATTCCCTAAGTTACATCAT

T T C
GCGTTCTTTGTGGGTGACATTTTAACAAAACGTGATTGTCTGAGGTTACAAACCTTGGCAGAAAATTGCCGTATTGTTAATATGTACGGTACCACTGAAACACAGCGTGCAGTTTCTTATTTCGAAGTTAAATCAAAAAA

G C C
TGACGATCCAAACTTTTTGAAAAAATTGAAAGATGTCATGCCTGCTGGTAAAGGTATGTTGAACGTTCAGCTACTAGTTGTTAACAGGAACGATCGTACTCAAATATGTGGTATTGGCGAAATAGGTGAGATTTATGTTC

A C
GTGCAGGTGGTTTGGCCGAAGGTTATAGAGGATTACCAGAATTGAATAAAGAAAAATTTGTGAACAACTGGTTTGTTGAAAAAGATCACTGGAATTATTTGGATAAGGATAATGGTGAACCTTGGAGACAATTCTGGTT

T A T
AGGTCCAAGAGATAGATTGTACAGAACGGGTGATTTAGGTCGTTATCTACCAAACGGTGACTGTGAATGTTGCGGTAGGGCTGATGATCAAGTTAAAATTCGTGGGTTCAGAATCGAATTAGGAGAAATAGATACGCA

T A T
CATTTCCCAACATCCATTGGTAAGAGAAAACATTACTTTAGTTCGCAAAAATGCCGACAATGAGCCAACATTGATCACATTTATGGTCCCAAGATTTGACAAGCCAGATGACTTGTCTAAGTTCCAAAGTGATGTTCCAA

G G T
AGGAGGTTGAAACTGACCCTATAGTTAAGGGCTTAATCGGTTACCATCTTTTATCCAAGGACATCAGGACTTTCTTAAAGAAAAGATTGGCTAGCTATGCTATGCCTTCCTTGATTGTGGTTATGGATAAACTACCAT
