# Supplementary figures and images for: DNA strand-exchange patterns associated with double-strand break-induced and spontaneous mitotic crossovers in Saccharomyces cerevisiae
Source: PLoS Genet. 2018 Mar 26;14(3):e1007302. doi: 10.1371/journal.pgen.1007302 (PMC5886692; doi:10.1371/journal.pgen.1007302)

DSB two-sided hetDNA (n=61)

*expected* 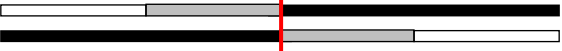

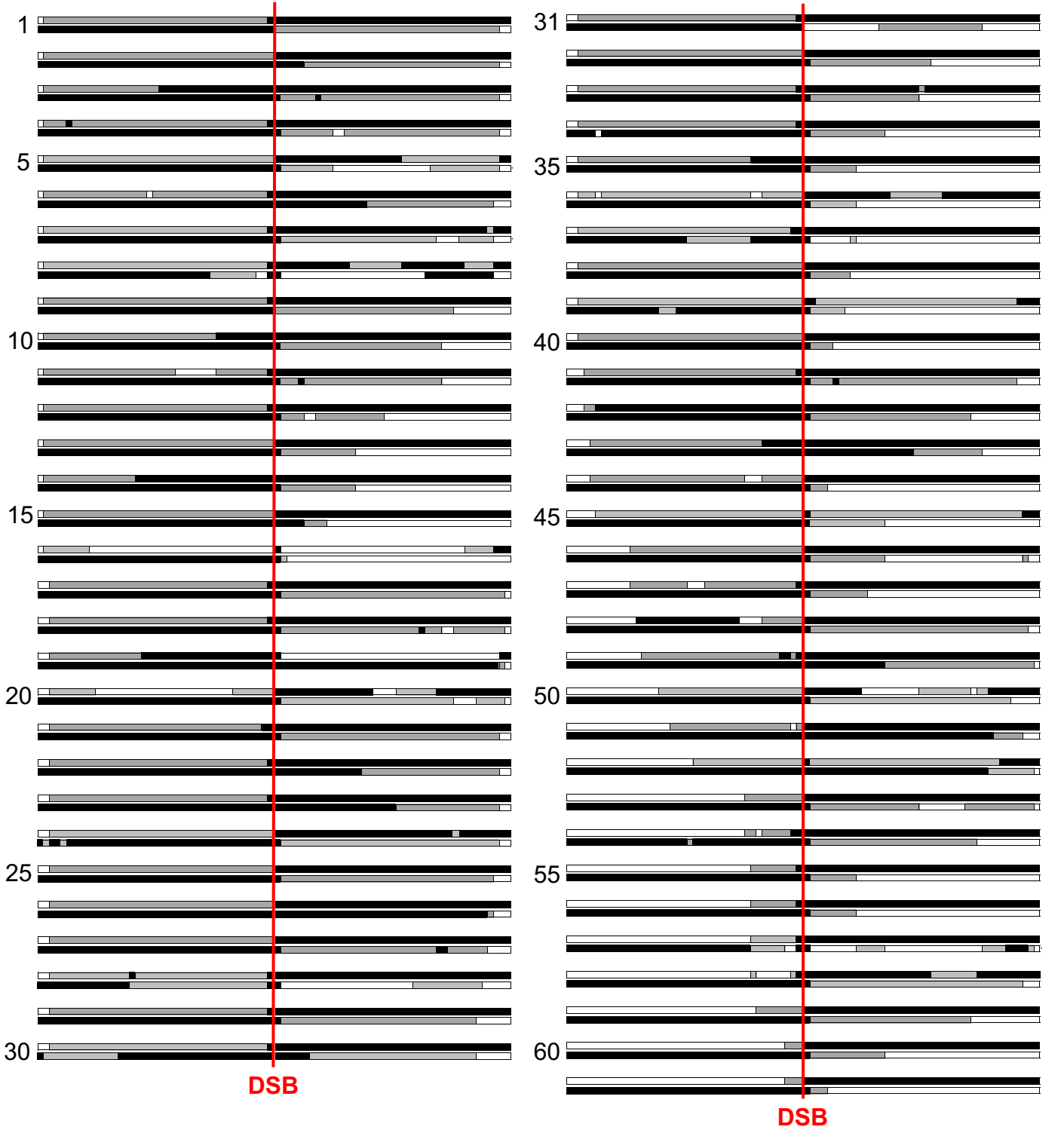

Supplement: S2 Fig — The lys2Δ5′Δ3′ and LYS2 alleles for each CO event in Fig 3 are shown stacked rather than side-by-side to allow visualization of hetDNA and gene conversion transitions. The lys2Δ5′Δ3′ is at the top of each pair of products and the LYS2 allele at the bottom. Events are aligned vertically based on the upstream hetDNA length so that their order is as in Fig 3. The vertical red line indicates the position of the initiating DSB. (PDF) [file pgen.1007302.s003.pdf]

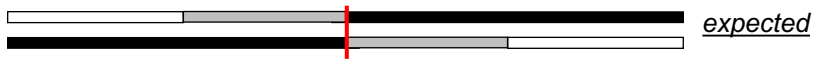

### A. One-sided hetDNA (n=35)

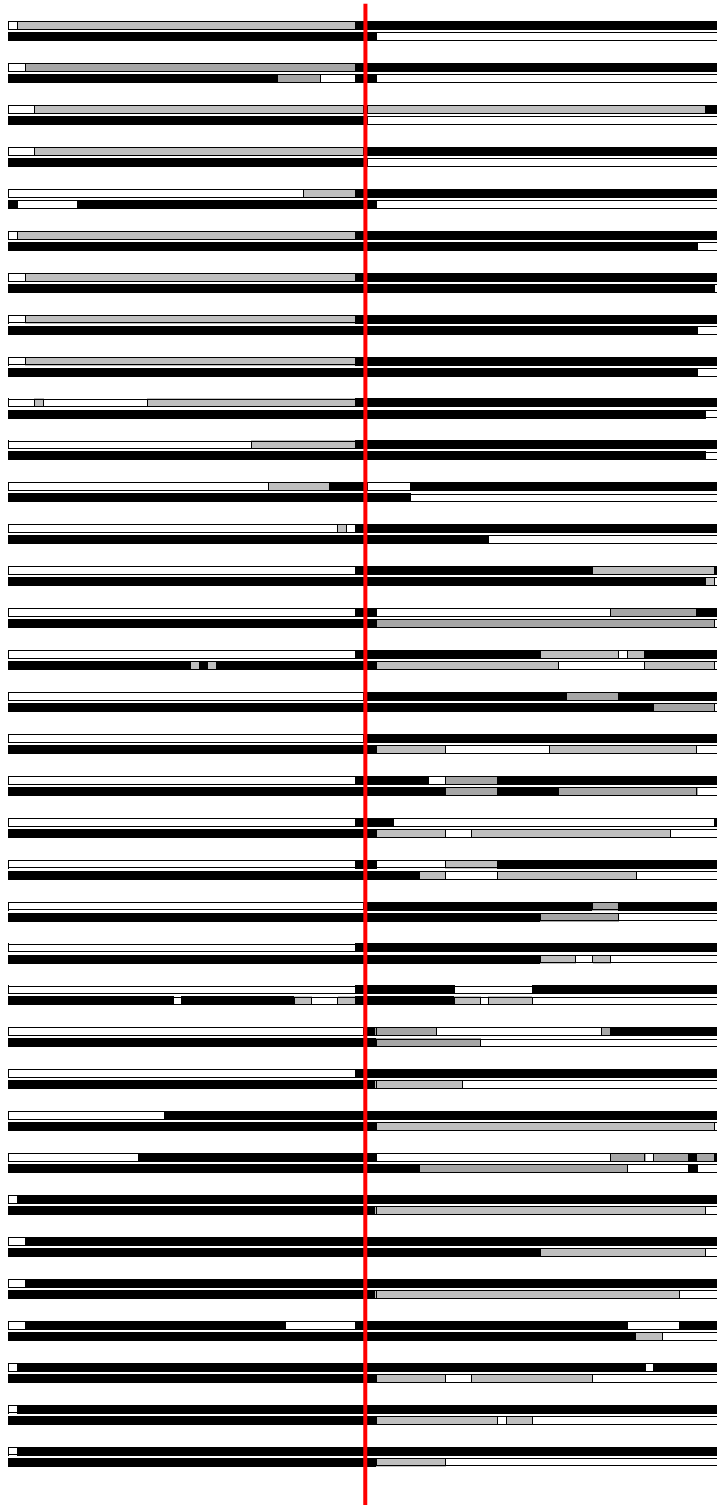

### B. One-sided hetDNA with displaced transition (n=3)

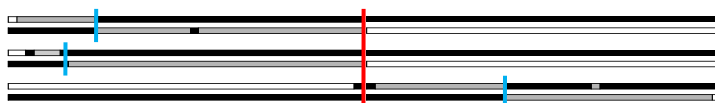

Supplement: S4 Fig — The lys2Δ5′Δ3′ and LYS2 alleles for each CO event in Fig 5 are shown stacked rather than side-by-side, and are in the same order as in Fig 5. The lys2Δ5′Δ3′ is at the top of each pair of products and the LYS2 allele at the bottom. The vertical red line indicates the position of the initiating DSB. (A) hetDNA on either the left or right side of the DSB. (B) The transition of asymmetric hetDNA from one product to the other is displaced from the DSB and is indicated by the blue vertical line. “n” is the number of events with the relevant hetDNA pattern. (PDF) [file pgen.1007302.s005.pdf]

**A. Donor-only hetDNA (n=7)**

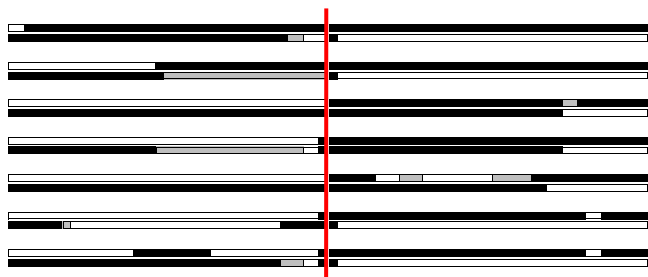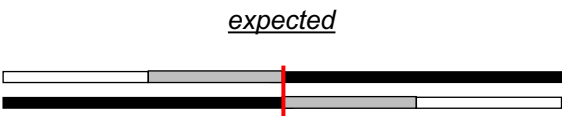

**B. No hetDNA (n=60)**

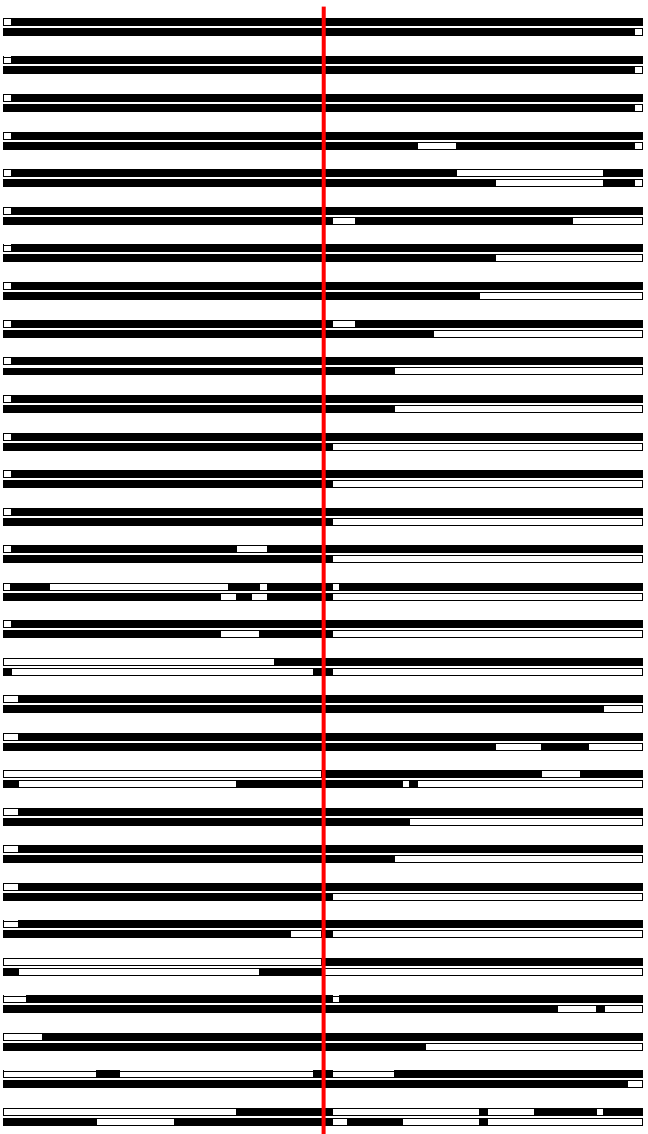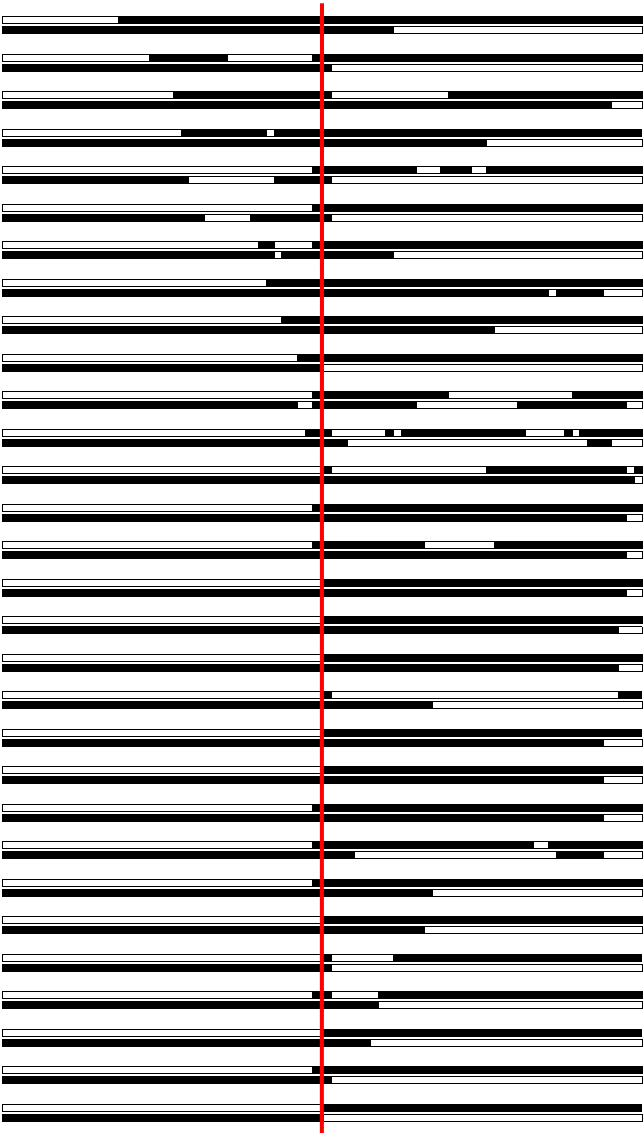

Supplement: S5 Fig — CO products for each event are stacked; the lys2Δ5′Δ3′ allele is on the top and the LYS2 allele on the bottom of each pair. (A) hetDNA was confined to the donor allele. (B) hetDNA was absent in CO products, but gene conversion tracts were present. The vertical red line indicates the position of the initiating DSB. “n” is the number of events with the relevant hetDNA pattern. (PDF) [file pgen.1007302.s006.pdf]

**A. No hetDNA (n=55)**

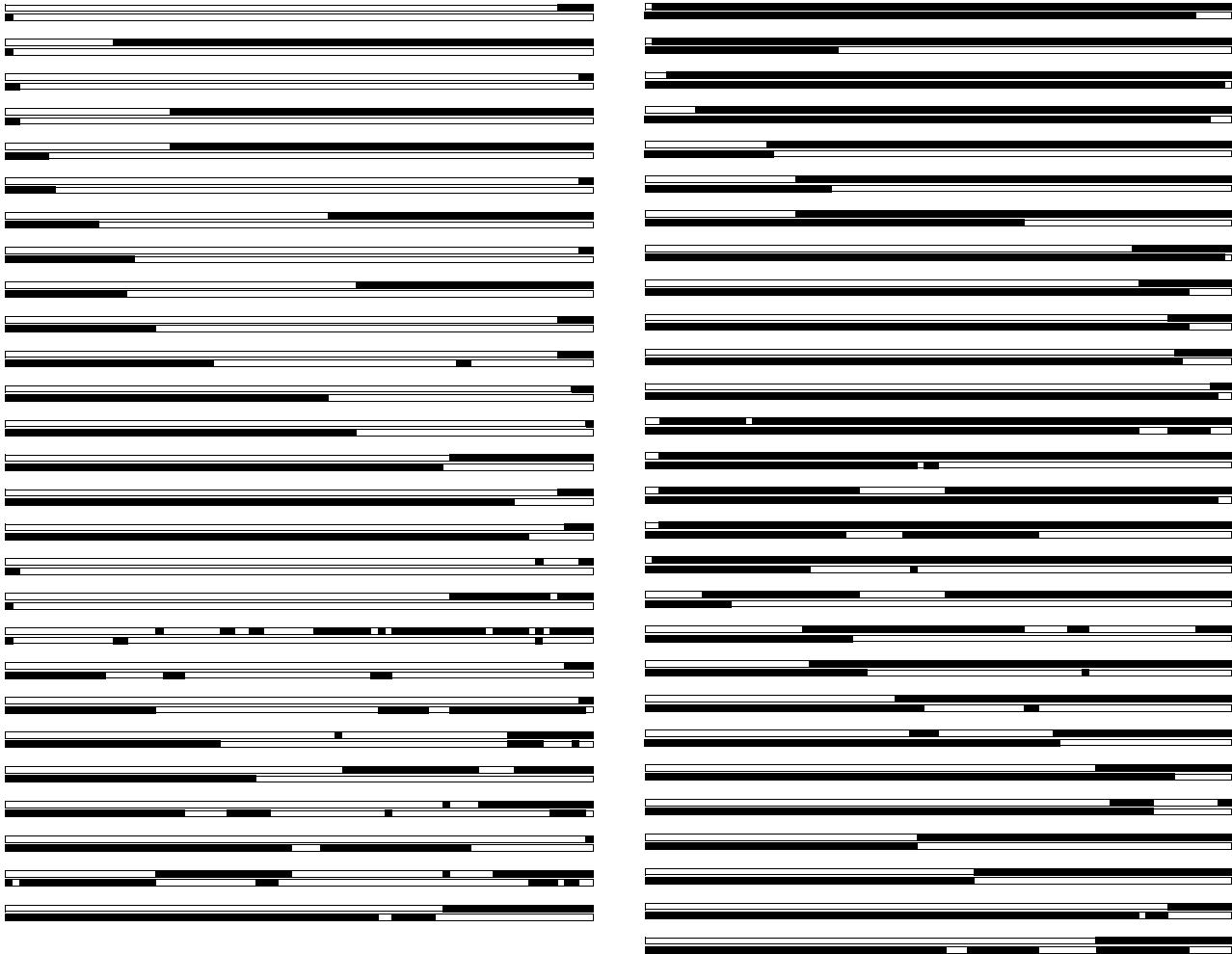

**B. hetDNA in one product (n=21)**

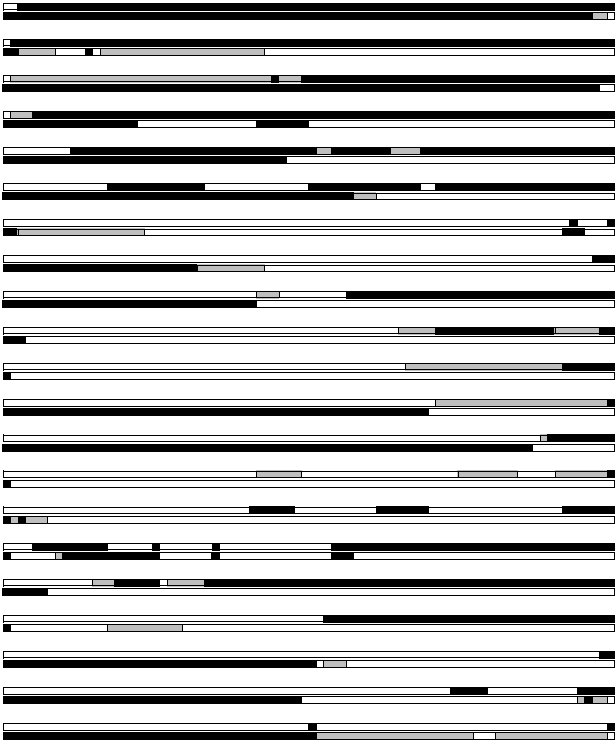

**C. Patchy hetDNA pattern (n=8)**

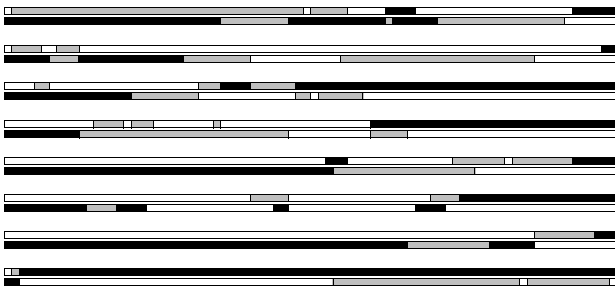

Supplement: S6 Fig — All events were isolated in a wild-type (TSA1) background. CO products for each event are stacked; the lys2Δ5′Δ3′ allele is on the top and the LYS2 allele on the bottom of each pair. (A) CO products with no detectable hetDNA. (B) CO events with hetDNA confined to only one allele. (C) CO events with hetDNA in both alleles, but with a “patchy” pattern that did not match that predicted for a DSB/nick/gap-induced COs (see Fig 1). (PDF) [file pgen.1007302.s007.pdf]

**A. No hetDNA (n=62)**

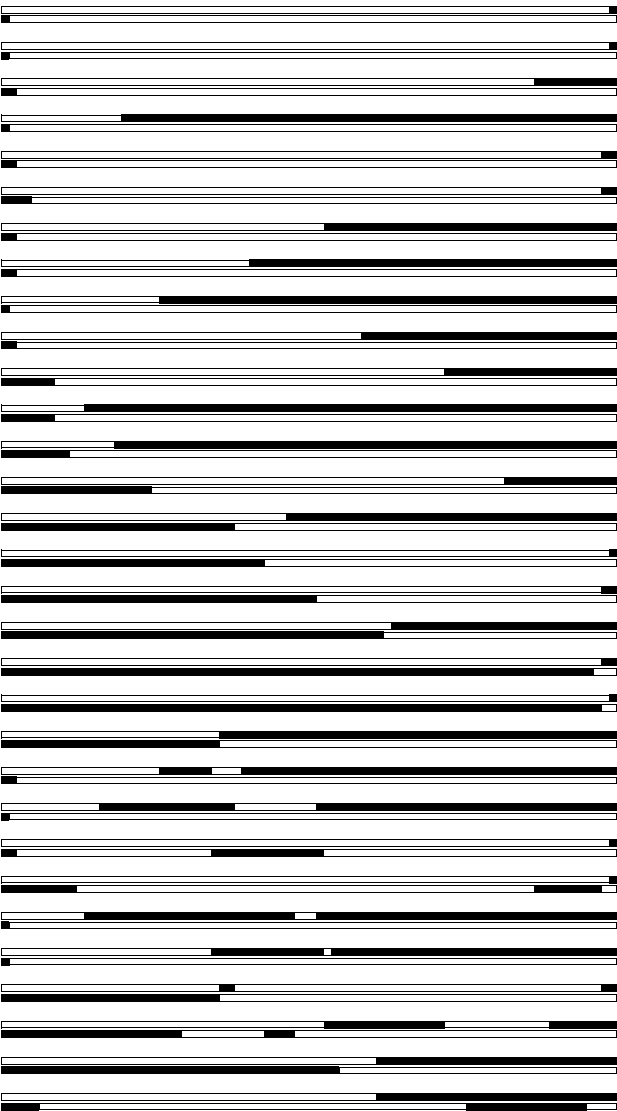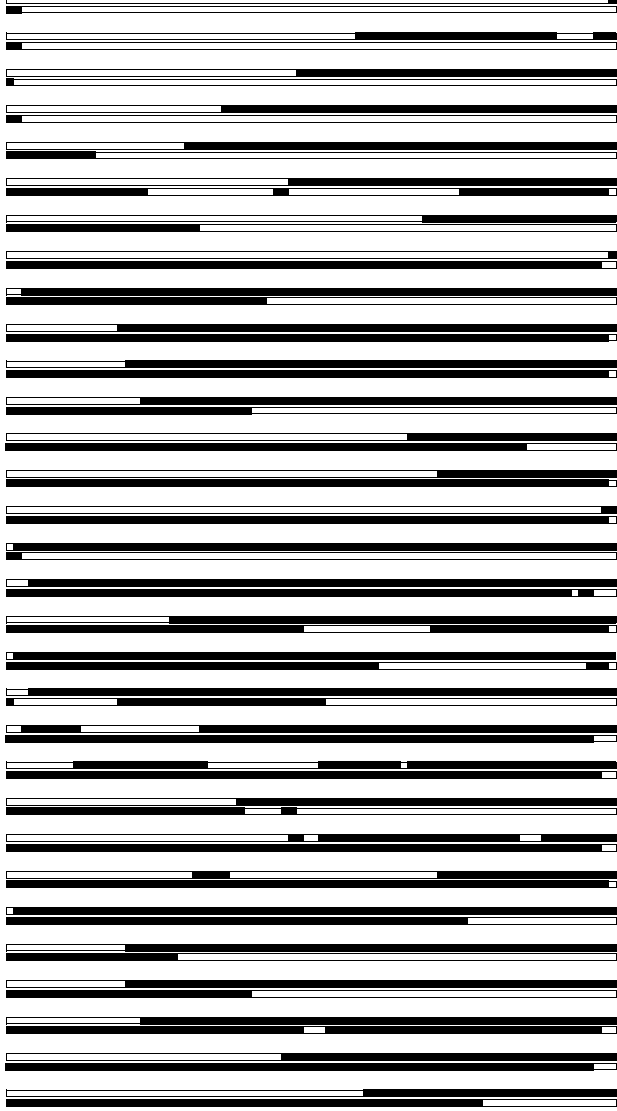

**B. hetDNA in one product (n=14)**

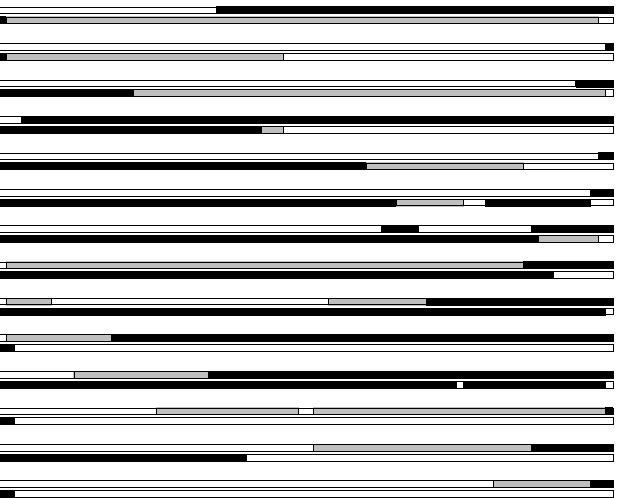

**C. Patchy hetDNA pattern (n=10)**

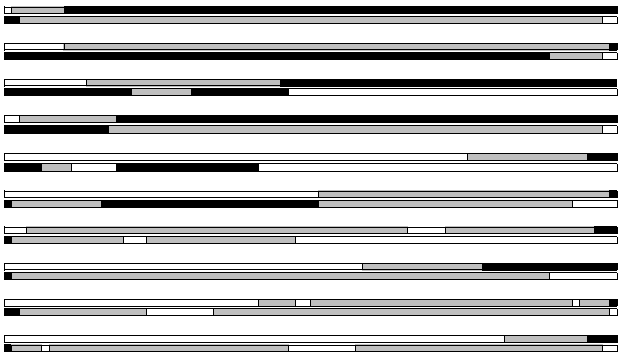

Supplement: S7 Fig — All events were isolated in a tsa1Δ background. CO products for each event are stacked; the lys2Δ5′Δ3′ allele is on the top and the LYS2 allele on the bottom of each pair. (A) CO products with no detectable hetDNA. (B) CO events with hetDNA confined to only one allele. (C) CO events with hetDNA in both alleles, but with a “patchy” pattern that did not match that predicted for a DSB/nick/gap-induced CO (see Fig 1). (PDF) [file pgen.1007302.s008.pdf]
