# Supplementary material for: DNA strand-exchange patterns associated with double-strand break-induced and spontaneous mitotic crossovers in Saccharomyces cerevisiae
Source: PLoS Genet. 2018 Mar 26;14(3):e1007302. doi: 10.1371/journal.pgen.1007302 (PMC5886692; doi:10.1371/journal.pgen.1007302)
Supplement: S3 Fig — Red and black lines represent the lys2Δ5′ and lys2Δ3′ alleles, respectively. Arrowheads mark the 3′ ends of DNA strands and yellow boxes the hetDNA. Black and gray triangles indicate sites of HJ cleavage. (A) Nick-directed HJ cleavage (cleavage of the strand with the same polarity as the nicked strand; black triangles) occurs before HJs are fully ligated. This generates CO products with hetDNA always upstream and downstream of the DSB in the lys2Δ5’Δ3’ and LYS2 alleles, respectively. The same pattern is produced regardless of which broken end invades the repair template. (B) Random cleavage occurs when the HJs are fully ligated. Alternative cleavage sites that produce CO products are represented by the black and gray triangles. Cleavage at the positions of the black triangles produces the same hetDNA pattern as nick-directed cleavage, while cleavage at the positions of the gray triangles reverses the hetDNA positions relative to the DSB. (PDF) [file pgen.1007302.s004.pdf]

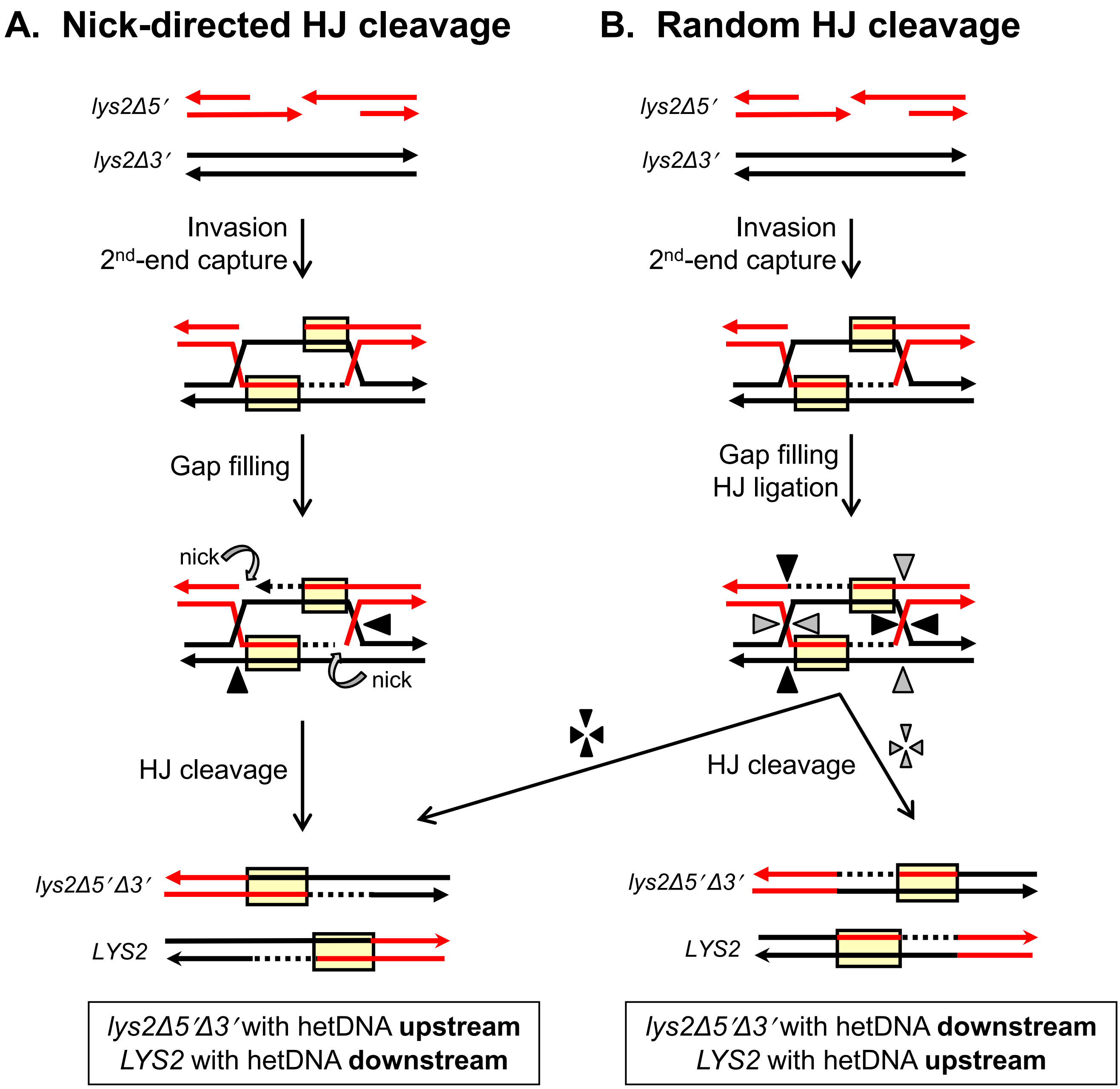
A. Nick-directed HJ cleavage
B. Random HJ cleavage
lys2Δ5′
lys2Δ3′
Invasion
2nd-end capture
Gap filling
nick
HJ cleavage
lys2Δ5′Δ3′
LYS2
lys2Δ5′Δ3′ with hetDNA upstream
LYS2 with hetDNA downstream
lys2Δ5′
lys2Δ3′
Invasion
2nd-end capture
Gap filling
HJ ligation
HJ cleavage
lys2Δ5′Δ3′
LYS2
lys2Δ5′Δ3′ with hetDNA downstream
LYS2 with hetDNA upstream
